# Molecular Characterization and Inhibition of a Novel Stress-Induced Mitochondrial Protecting Role for Misfolded TrkAIII in Human SH-SY5Y Neuroblastoma Cells

**DOI:** 10.3390/ijms25105475

**Published:** 2024-05-17

**Authors:** Lucia Cappabianca, Marianna Ruggieri, Michela Sebastiano, Maddalena Sbaffone, Ilaria Martelli, Pierdomenico Ruggeri, Monica Di Padova, Antonietta Rosella Farina, Andrew Reay Mackay

**Affiliations:** Department of Biotechnological and Applied Clinical Sciences, University of L’Aquila, Via Vetoio, L’Aquila 67100, Italy; luciaannamaria.cappabianca@univaq.it (L.C.); marianna.ruggieri@graduate.univaq.it (M.R.); michela.sebastiano@graduate.univaq.it (M.S.); maddalena.sbaffone@graduate.univaq.it (M.S.); ilaria.martelli@graduate.univaq.it (I.M.); pierdomenicoruggeri@gmail.com (P.R.); monica.dipadova@univaq.it (M.D.P.); antonietta.farina@univaq.it (A.R.F.)

**Keywords:** TrkAIII, stress resistance, mitochondria, integrated stress response, Grp78, Ca^2+^-calmodulin, adenosine ribosylating factor, mitochondrial Ca^2+^ uniporter, ROS, PI3K, Akt

## Abstract

Pediatric neuroblastomas (NBs) are heterogeneous, aggressive, therapy-resistant embryonal tumors that originate from cells of neural crest origin committed to the sympathoadrenal progenitor cell lineage. Stress- and drug-resistance mechanisms drive post-therapeutic relapse and metastatic progression, the characterization and inhibition of which are major goals in improving therapeutic responses. Stress- and drug-resistance mechanisms in NBs include alternative *TrkAIII* splicing of the neurotrophin receptor tropomyosin-related kinase A (*NTRK1/TrkA*), which correlates with post-therapeutic relapse and advanced-stage metastatic disease. The TrkAIII receptor variant exerts oncogenic activity in NB models by mechanisms that include stress-induced mitochondrial importation and activation. In this study, we characterize novel targetable and non-targetable participants in this pro-survival mechanism in TrkAIII-expressing SH-SY5Y NB cells, using dithiothreitol (DTT) as an activator and a variety of inhibitors by regular and immunoprecipitation Western blotting of purified mitochondria and IncuCyte cytotoxicity assays. We report that stress-induced TrkAIII misfolding initiates this mechanism, resulting in Grp78, Ca^2+^-calmodulin, adenosine ribosylating factor (Arf) and Hsp90-regulated mitochondrial importation. TrkAIII imported into inner mitochondrial membranes is cleaved by Omi/high temperature requirement protein A2 (HtrA2) then activated by a mechanism dependent upon calmodulin kinase II (CaMKII), alpha serine/threonine kinase (Akt), mitochondrial Ca^2+^ uniporter and reactive oxygen species (ROS), involving inhibitory mitochondrial protein tyrosine phosphatase (PTPase) oxidation, resulting in phosphoinositide 3 kinase (PI3K) activation of mitochondrial Akt, which enhances stress resistance. This novel pro-survival function for misfolded TrkAIII mitigates the cytotoxicity of mitochondrial Ca^2+^ homeostasis disrupted during integrated stress responses, and is prevented by clinically approved Trk and Akt inhibitors and also by inhibitors of 78kDa glucose regulated protein (Grp78), heat shock protein 90 (Hsp90), Ca^2+^-calmodulin and PI3K. This identifies Grp78, Ca^2+^-calmodulin, Hsp90, PI3K and Akt as novel targetable participants in this mechanism, in addition to TrkAIII, the inhibition of which has the potential to enhance the stress-induced elimination of TrkAIII-expressing NB cells, with the potential to improve therapeutic outcomes in NBs that exhibit TrkAIII expression and activation.

## 1. Introduction

Pediatric neuroblastomas (NBs) are heterogeneous, aggressive, therapy-resistant embryonal tumors that originate from cells of neural crest origin and in particular neuroblasts committed to the sympathoadrenal progenitor cell lineage. NBs represent the most common extracranial pediatric solid tumor, can develop anywhere along the sympathetic chain, are more frequent in the abdomen and adrenal medulla and account for ≈15% of cancer-related pediatric deaths. NB clinical heterogeneity ranges from spontaneous regression to aggressive metastatic disease associated with therapeutic resistance. Low- and intermediate-risk NBs exhibit cure rates of 80–90%, which despite intensive therapeutic regimens drop to <50% for high-risk disease, with <10% survival associated with relapsed recurrent disease, highlighting the need to improve therapeutic approaches [1,2,3].

Stress-adaptation mechanisms and the acquisition of drug resistance underpin aggressive tumor cell selection within tumor microenvironments and increase the probability of post-therapeutic relapse and disease progression [4,5]. This makes the characterization and inhibition of stress-adaptation and drug-resistance mechanisms major goals in improving and prolonging therapeutic responses and enhancing patient survival [4,5]. Within this context, we previously reported a pro-survival stress-adaptation and drug-resistance mechanism in human neuroblastoma (NB), characterized by alternative *TrkAIII* splicing of the neurotrophin receptor tropomyosin-related kinase gene *NTRK1/TrkA,* which results in the expression of the oncogenic TrkAIII receptor variant [6,7]. The alternative *TrkAIII* splice variant (GeneBank: OP866787.1) was originally discovered in human NBs and correlated with advanced-stage metastatic disease and post-therapeutic relapse [6,7,8]. *TrkAIII* mRNA is characterized by in-frame *TrkA* cassette exons 6-7 and 9 skipping and is expressed as a variant oncoprotein devoid of the extracellular D4 IG-C1 domain and several N-glycosylation sites, encoded within skipped exons 6 and 7, required for fully spliced TrkA receptor cell surface expression and prevention of spontaneous receptor activation. As a consequence of these omissions, the TrkAIII receptor variant does not exhibit cell surface expression but re-localizes to pre-Golgi membranes, centrosomes and mitochondria, where it exhibits cell cycle and stress-regulated ligand-independent activation, resulting in oncogenic activity (NIH3T3 cell transformation and promotion of primary and metastatic tumorigenicity in NB models) [6,7,9,10,11]. In this respect, TrkAIII is the pathophysiological equivalent of an engineered D4 domain-deleted TrkA oncogene [8]. Furthermore, TrkAIII also exhibits similar oncogenic behaviour in an in vivo NB model [6] to the TrkA fusion oncogene, Trk-T3 [12]. This characterizes TrkAIII as an oncogenic splice variant equivalent to TrkA fusion oncogenes [12,13], the potential importance of which is bolstered by reports that alternative splicing is a hallmark of cancer and represents an important alternative oncogene and oncogenic pathway activation mechanism in tumors that exhibit low mutation rates, which include NBs [14,15,16,17].

In previous studies, we reported that alternative *TrkAIII* splicing is promoted by hypoxia in NB cells, normal neural crest progenitors and neural stem cells [6] and in NB cells is also promoted by agents that cause nutrient, redox and Ca^2+^ stress and by SV40 polyomavirus Large T antigen [6,7,18]. In stable transfected TrkAIII-expressing SH-SY5Y NB cells, intracellular cell cycle-regulated ligand-independent TrkAIII activation induces pro-survival PI3K/Akt signaling, enhances Bcl-xL, Mcl-1 and SOD2 expression, promotes a pro-angiogenic MMP-9/VEGF/Tsp1 expression equilibrium and a more anaplastic stem cell-like phenotype, induces centrosome amplification and modifies the endoplasmic reticulum (ER) stress response [6,7,19]. Under conditions of severe ER stress, induced by DTT, the Ca^2+^ ionophore A23187 or the SERCA Ca^2+^ pump inhibitor thapsigargin, TrkAIII alters its behaviour and is imported into inner mitochondrial membranes (IMMs), where it is cleaved to a 48 kDa carboxyl (C-) terminus fragment by the inner mitochondrial membrane space (IMS) serine protease Omi/HtrA2 and activated. Stress-induced intra-mitochondrial TrkAIII activation results in PDHK tyrosine phosphorylation, glycolytic adaptation and enhanced resistance to stress-induced death [20], placing mitochondria at the heart of TrkAIII involvement in resistance to cytotoxic disruption of Ca^2+^ homeostasis.

Mitochondria are not only central to eukaryotic metabolism but also regulate Ca^2+^ homeostasis, cell survival and death [21,22,23]. These organelles are structurally and functionally linked to the ER by mitochondrial-associated membranes (MAMs) [24,25], which are involved in integrating ER stress responses with mitochondria. ER stress responses in tumor cells are induced by conditions within the tumor microenvironment (hypoxia, cyclic hypoxia and reoxygenation and nutrient deprivation, etc.), and their integration with mitochondria provides additional routes for enhancing survival and restoring proteostasis [4,5]. These routes include mitochondrial importation of Ca^2+^ released from the ER, for temporary storage and buffering, and misfolded glycosylated proteins for degradation by mitochondrial quality control and proteostasis IMS (Omi/HtrA2), IMM and matrix (YME1L1, OMA1, PARL, CLpP and LonP m-AAA and i-AAA) proteases [26,27,28,29,30,31,32]. Stress-induced disruption of ER Ca^2+^ homeostasis causes the release of ER Ca^2+^, which is imported into mitochondria via MAMs for transient storage and buffering in order to mitigate the potential cytotoxicity of surges in Ca^2+^ movement across intracellular membranes [21,22,23,24,25,26,27]. This, however, may lead to cytotoxic mitochondrial Ca^2+^ overload, resulting in an increase in mitochondrial matrix Ca^2+^ levels that can result in aberrant opening of the mitochondrial permeability transition pore (mptp) and a cytotoxic burst in ROS production, which, when combined, induce the collapse of mitochondrial membrane potential, mitochondrial membrane permeability and apoptosis [4,33,34,35,36,37]. Survival adaptations that reduce the cytotoxicity of tumor cell mitochondrial Ca^2+^ homeostasis disruption by tumor microenvironmental conditions are likely to be critical in selecting highly resistant sub-populations that underpin NB progression and post-therapeutic relapse. The stressful effects of the tumor microenvironment on NB cells can be mimicked in vitro using the reducing agent DTT, calcium ionophore A23187 or the SERCA Ca^2+^ pump inhibitor thapsigargin, all of which disrupt Ca^2+^ homeostasis, promote protein misfolding and activate integrated stress responses with cytotoxic consequences [7,19,20,33,34,35,36,37]. In TrkAIII-expressing SH-SY5Y cells, these agents promote mitochondrial TrkAIII importation and cleavage activation, resulting in enhanced resistance to stress-induced death [20].

In the current study, in light of the correlation between alternative TrkAIII splicing, advanced-stage metastatic disease and post-therapeutic relapse in NBs [6,8], and the significance of mitochondrial TrkAIII translocation and activation in NB cell stress resistance [20], we undertook a more detailed investigation of the molecular requirements and participants involved in stress-induced mitochondrial TrkAIII importation and cleavage activation, with the goal of identifying novel targets, the inhibition of which enhances stress-induced elimination of TrkAIII-expressing NB cells.

## 2. Results

### 2.1. DTT Induces Mitochondrial TrkAIII Re-Localization, Cleavage Activation, Increases Mitochondrial PTPase Oxidation and Enhances Resistance to DTT-Induced Death

In order to assess the effects of DTT-induced stress on TrkAIII behaviour, mitochondrial translocation and activation, untreated and DTT-treated TrkAIII SH-SY5Y cells were compared by indirect immunofluorescence (IF), using anti-TrkA and anti-Y490 phosphorylated TrkA antibodies that recognize TrkAIII [6], and MitoTracker mitochondrial staining dye.

**Figure 1:**
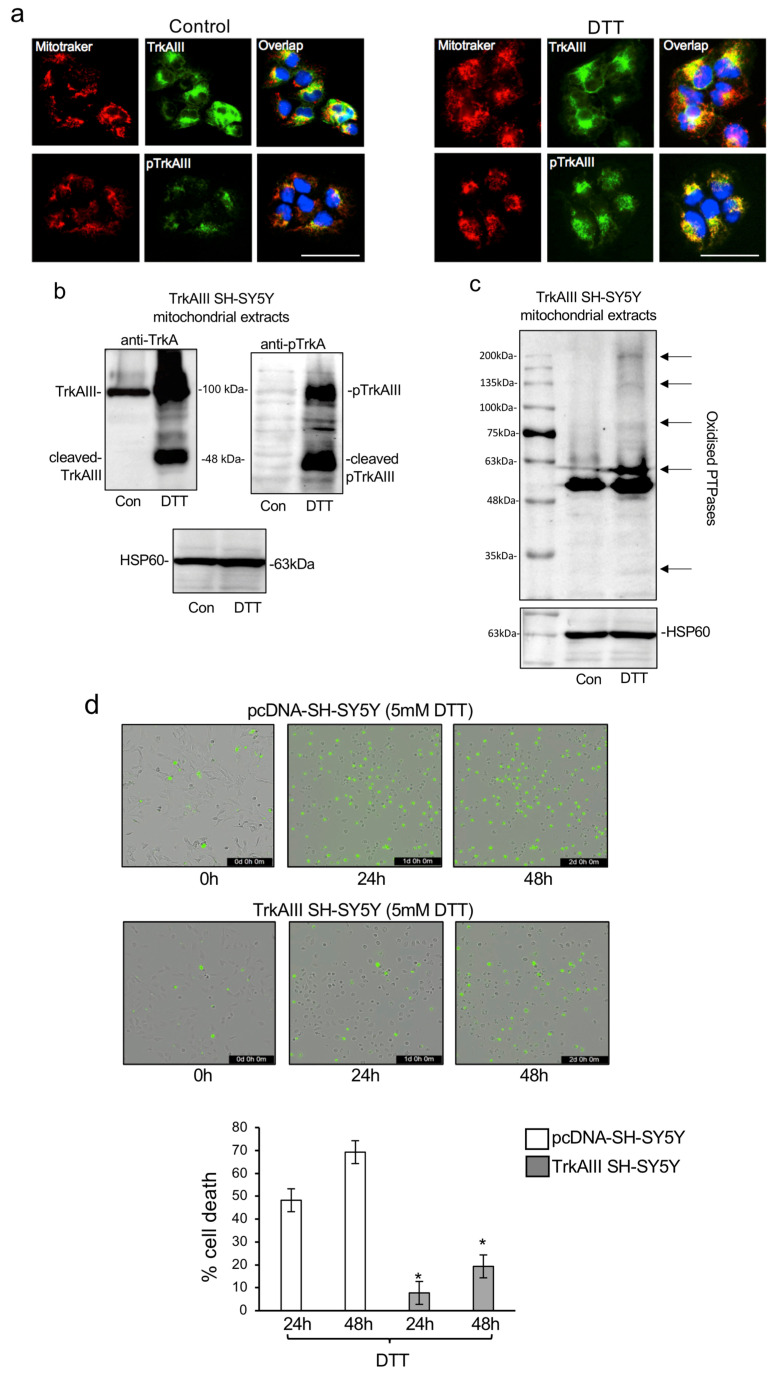
(**a**) Indirect IFs demonstrating increased overlapping (yellow/orange) immunoreactivity for TrkA (green) and MitoTracker-labeled mitochondria (red) (upper panels) and Y490 phosphorylated TrkAIII (pTrkAIII, green) and MitoTracker-labeled mitochondria (red) (lower panels) in DTT-treated (5 mM for 6 h) TrkAIII SH-SY5Y cells compared to untreated controls (control). DAPI stained nuclei are blue. (bar = 50 μm). (**b**) Western blots demonstrating TrkAIII cleavage and Y674/5 phosphorylation in mitochondria (50 mg) from DTT-treated (5 mM for 6 h) TrkAIII SH-SY5Y cells compared to untreated TrkAIII SH-SY5Y controls. (**c**) Western blots demonstrating increased PTPase oxidation (arrows) in mitochondria (50 mg) from DTT-treated TrkAIII SH-SY5Y cells (5 mM for 6 h) compared to untreated controls (Con). (**d**) Phase contrast images merged with green fluorescence and histogram demonstrating significant differences (* *p* < 0.0001) in pcDNA-SH-SY5Y and TrkAIII SH-SY5Y percentage cell death at 24 h and 48 h, induced by 5 mM DTT.

IF detected a notable increase in overlapping (yellow/orange) TrkAIII and Y490 phosphorylated TrkAIII immunoreactivity (green) with MitoTracker-stained (red) mitochondria in TrkAIII SH-SY5Y cells treated for 6 h with 5 mM DTT, compared to untreated TrkAIII SH-SY5Y cell controls (Figure 1a). These data show that DTT alters TrkAIII behaviour, increasing its association with mitochondria in phosphorylated form.

To assess DTT influence on the status and activation of TrkAIII, Western blots were performed on purified mitochondrial extracts from untreated and DTT-treated TrkAIII SH-SY5Y cells. Western blots detected non-phosphorylated 100 kDa TrkAIII in mitochondrial extracts from untreated TrkAIII SH-SY5Y cells and in mitochondrial extracts from TrkAIII SH-SY5Y cells treated for 6 h with DTT, detected an increase in TrkAIII levels and induction of TrkAIII cleavage to a 48 kDa C-terminus fragment and the Y674/5 phosphorylation of uncleaved TrkAIII and cleaved 48 kDa TrkAIII C-terminus fragment (Figure 1b). These data show that DTT increases TrkAIII association with mitochondria and promotes mitochondrial TrkAIII cleavage and phosphorylation, consistent with activation [6].

Since oxidative inhibition of PTPases [38] facilitates TrkAIII activation [20], mitochondrial extracts from untreated and DTT-treated TrkAIII SH-SY5Y cells were also examined for changes in the levels of oxidized PTPases by Western blotting. Increased (arrows) levels of oxidized PTPases were detected in mitochondria from DTT-treated (5 mM for 6 h) compared to untreated TrkAIII SH-SY5Y cells (Con) (Figure 1c), consistent oxidative PTPase inhibitory conditions.

To evaluate the relative cytotoxicity of DTT to TrkAIII-expressing (TrkAIII SH-SY5Y) and non-expressing control pcDNA-SH-SY5Y cells, IncuCyte cytotoxicity assays were performed, employing IncuCyte^®^ Cytotox Green Dye to detect cell membrane integrity disruption in real time to quantify cell death. In duplicate assays, repeated three times, treatment of pcDNA-SH-SY5Y cells with DTT induced a mean (±s.d.) percentage cell death of 48.3 (±4.2)% at 24 h and 69.2 (±4.1)% at 48 h. In contrast, DTT treatment of TrkAIII SH-SY5Y cells induced significantly lower 7.8 ± 1% cell death at 24 h and 19.3 ± 1.8% cell death at 48 h (*p* < 0.0001 for comparisons between DTT-induced pcDNA-SH-SY5Y and TrkAIII death at 24 h and for DTT-induced pcDNA-SH-SY5Y and TrkAIII death at 48 h) (Figure 1d).

These data confirm and extend our previous report [20] that DTT induces mitochondrial TrkAIII translocation and cleavage activation in association with an increase in mitochondrial PTPases oxidation, resulting in enhanced resistance to DTT-induced death.

### 2.2. DTT-Induced Mitochondrial Importation and Cleavage Activation of TrkAIII Involves Misfolding and Complexing with GRP78 and Ca^2+^-Calmodulin

**Figure 2:**
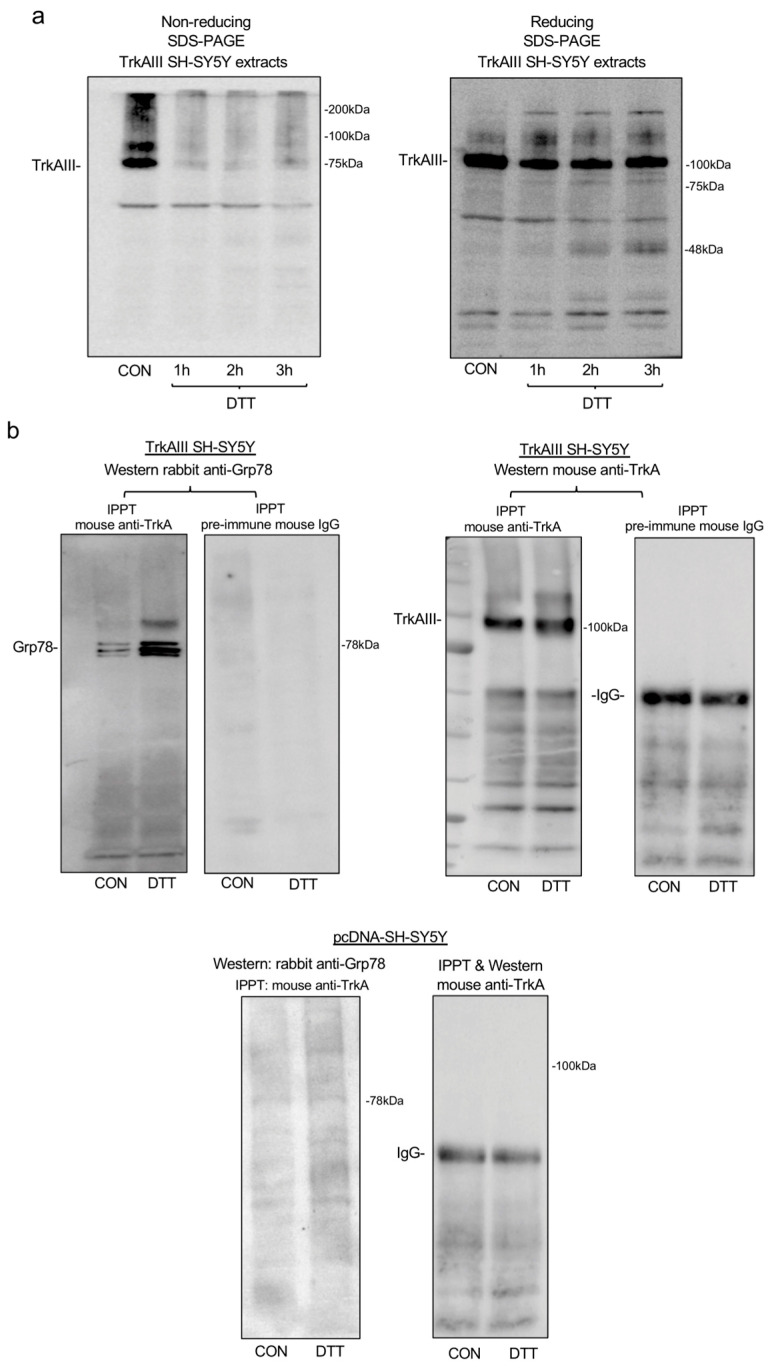

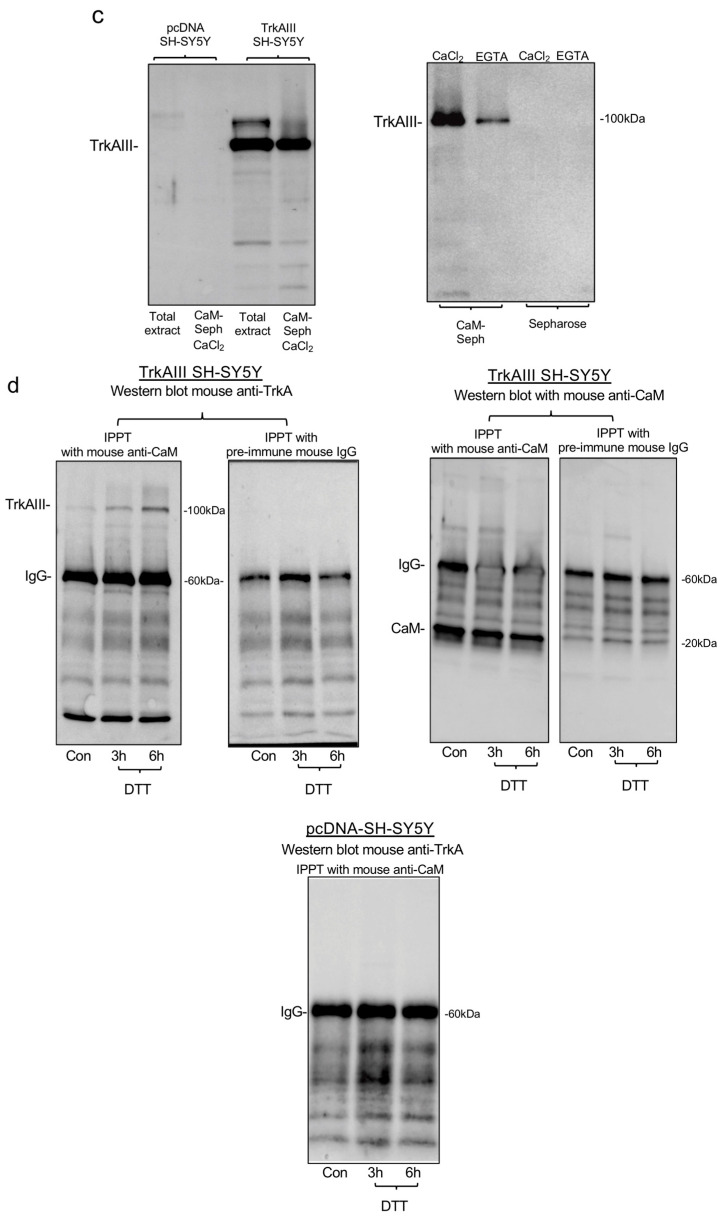
(**a**) Western blots demonstrating differences in TrkAIII immunoreactivity in untreated (CON) and DTT-treated (5 mM DTT for 1, 2 and 3 h) TrkAIII SH-SY5Y cell extracts (30 mg) under non-reducing and reducing conditions. (**b**) Co-immunoprecipitation Western blots demonstrating increased TrkAIII pulldown of Grp78 by anti-TrkA antibody in DTT-treated (5 mM for 6 h) TrkAIII SH-SY5Y cell extracts compared to untreated control extracts (CON) and similar levels of TrkAIII immunoprecipitated by anti-TrkA antibody but not by pre-immune mouse IgG in untreated (Con) and DTT-treated (5 mM for 6 h) TrkAIII SH-SY5Y cell extracts (500 μg), plus the absence of GRP78 or TrkA isoform pulldown by anti-TrkA antibody in pcDNA-SH-SY5Y cell extracts (500 μg). (**c**) Western blots of TrkAIII pulldown by calmodulin-conjugated Sepharose (CaM-Seph) in TrkAIII SH-SY5Y but not pcDNA-SH-SY5Y cell extracts (500 μg) in the presence of 150 mM CaCl_2_ (left panel) plus increased TrkAIII pulldown by calmodulin-conjugated Sepharose (CaM-Seph) in TrkAIII SH-SY5Y cell extracts (500 μg) in the presence of 150 μM CaCl_2_ compared to 5 mM EGTA (right panel), plus no TrkAIII pulldown by unconjugated Sepharose in TrkAIII SH-SY5Y cell extracts in the presence of 150 μM CaCl_2_ or 5 mM EGTA (right panel). (**d**) Co-immunoprecipitation Western blots demonstrating enhanced pulldown of TrkAIII by anti-calmodulin antibody (anti-CaM) but not by pre-immune mouse IgG in DTT-treated (5 mM for 3 and 6 h) but not untreated (Con) TrkAIII SH-SY5Y extracts (500 μg) (upper left panels) and calmodulin pulldown by anti-calmodulin antibody (anti-CaM) but not pre-immune IgG in untreated (Con) and DTT-treated (DTT) TrkAIII SH-SY5Y cell extracts (500 μg) (upper right panels), plus no pulldown of TrkAIII by anti-calmodulin antibody in pcDNA-SH-SY5Y extracts (500 μg).

To assess whether the altered behavior of TrkAIII induced by DTT treatment involved TrkAIII misfolding, TrkAIII immunoreactivity in cell extracts from untreated and DTT-treated TrkAIII SH-SY5Y cells was compared in non-reducing and reducing SDS-PAGE Western blots. Using an anti-TrkA antibody raised against the last 14 amino acids of the TrkA C-terminus, non-reducing SDS-PAGE Western blots detected a predominant ≈ 75 kDa TrkAIII immunoreactive species. In contrast, reducing SDS-PAGE Western blots detected a predominant ≈ 100 kDa TrkAIII immunoreactive species in untreated TrkAIII SH-SY5Y cell extracts (Figure 2a).

This difference can be explained by alterations in the non-reduced and reduced TrkAIII tertiary structure, which influence electrophoretic migration under denaturing SDS-PAGE conditions.

Following treatment with DTT for 1, 2 and 3 h, little change was detected in 100 kDa TrkAIII immunoreactive species detected by reducing SDS-PAGE Western blots. In contrast, 75 kDa TrkAIII immunoreactivity in non-reducing SDS-PAGE Western blots was almost completely lost at all time points (Figure 2a). These data indicate (i) that DTT treatment of TrkAIII SH-SY5Y cells results in reduction-sensitive masking of the TrkAIII C-terminus, recognized by the anti-TrkA antibody, in non-reducing Western blots and (ii) that exogenous DTT treatment of TrkAIII SH-SY5Y cells does not result in full TrkAIII reduction to 100 kDa, detected in reducing SDS-PAGE Western blots.

In order to further investigate the influence of DTT on TrkAIII folding and complexing, co-immunoprecipitation Western blot experiments were performed to assess changes in TrkAIII complexing with the misfolded protein-binding chaperone GRP78 [39].

In these experiments, TrkAIII immunoprecipitated from TrkAIII SH-SY5Y cells treated for 6 h with DTT pulled down substantially more Grp78 than TrkAIII immunoprecipitated from untreated TrkAIII SH-SY5Y cell extracts (Figure 2b). In pcDNA-SH-SY5Y cell extracts (500 μg), anti-TrkA antibody did not pull down Grp78 (Figure 2b).

These data indicate that DTT induces sufficient TrkAIII misfolding to promote complexing with the misfolded protein-binding chaperone GRP78.

In light of a report that fully spliced TrkA binds calmodulin in a Ca^2+^-dependent manner [40] and DTT deregulates Ca^2+^ homeostasis [37], we also assessed whether TrkAIII binds calmodulin and whether this interaction is influenced by DTT. In pulldown Western blot assays, Sepharose-conjugated calmodulin pulled down notably higher levels of TrkAIII from TrkAIII SH-SY5Y cell extracts in the presence of exogenous Ca^2+^ compared to TrkAIII levels pulled down by Sepharose-conjugated calmodulin in the presence of the Ca^2+^ chelator EGTA (Figure 2c). This confirms that TrkAIII also exhibits Ca^2+^-dependent complexing with calmodulin. In contrast, calmodulin Sepharose did not pull down TrkAIII from pcDNA-SH-SY5Y cell extracts (Figure 2c).

In co-immunoprecipitation Western blots, calmodulin immunoprecipitated by anti-calmodulin antibody from TrkAIII SH-SY5Y cells treated for 3 and 6 h with DTT, pulled down notably more TrkAIII than calmodulin immunoprecipitated from untreated TrkAIII SH-SY5Y cell extracts (Figure 2d). In contrast, the anti-calmodulin antibody did not pulldown TrkA isoforms from pcDNA-SH-SY5Y cell extracts (Figure 2d).

Overall, these data confirm that TrkAIII binds calmodulin in a Ca^2+^-dependent manner and show that DTT increases TrkAIII complexing with Ca^2+^-calmodulin in TrkAIII SH-SY5Y cells.

### 2.3. Mitochondrial TrkAIII Importation and Cleavage Activation Is Regulated by GRP78, Ca^2+^-Calmodulin and Arf1

Since DTT promotes mitochondrial importation (this study) and [20] promotes TrkAIII complexing with both GRP78 and calmodulin, and considering that adenosine ribosylating factor-1 (Arf1) regulates the formation and function of MAMs [41], required for ER to mitochondrial protein and Ca^2+^ transport [24,42,43], we investigated whether Grp78, Ca^2+^-calmodulin and Arf1 actively participate in DTT-induced mitochondrial TrkAIII translocation, cleavage and activation, using the Grp78 inhibitor HA-15 [44], the Ca^2+^-calmodulin W7 [45] and the Arf1 inhibitor brefeldin A (BfA) [46].

**Figure 3:**
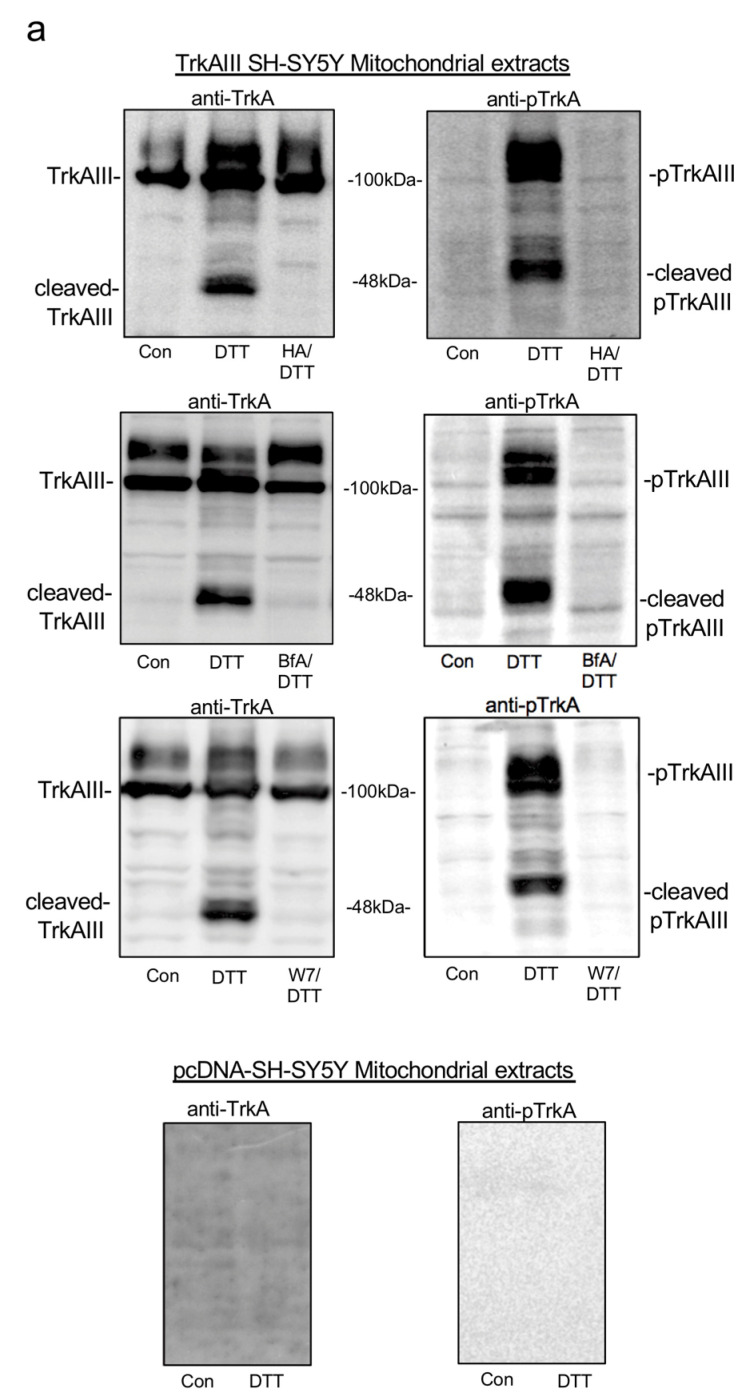

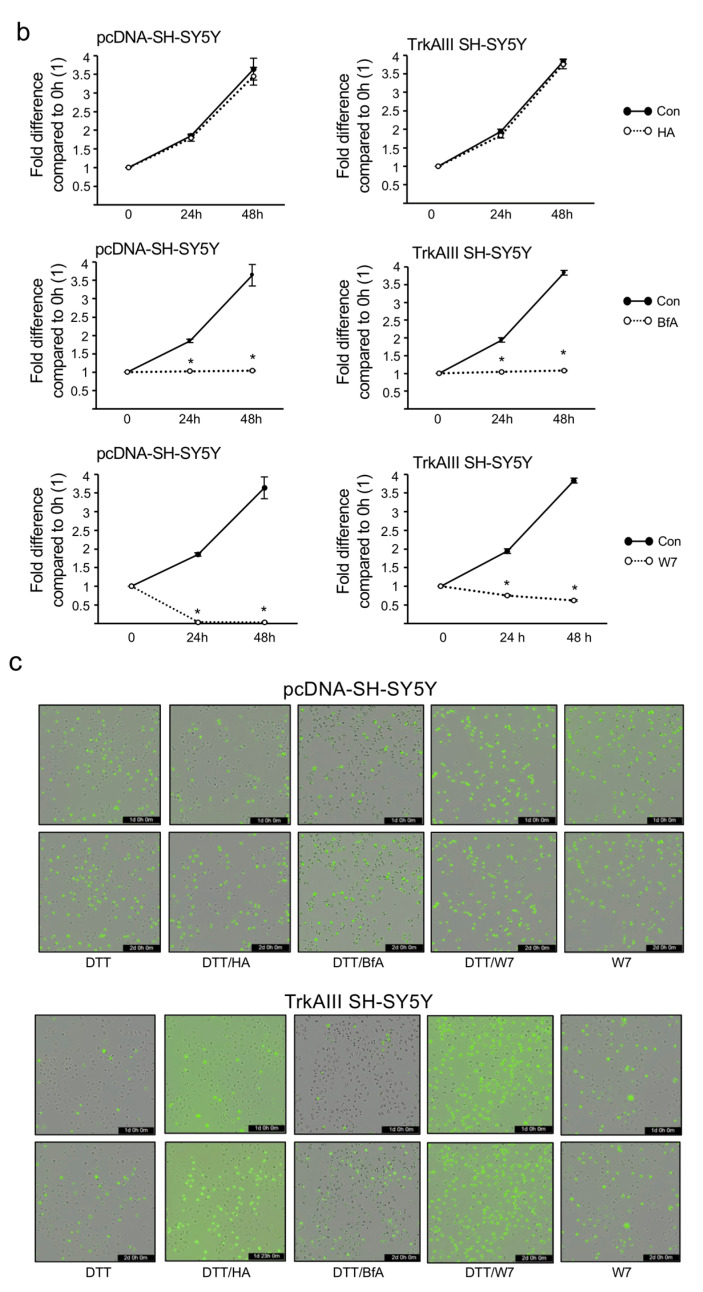

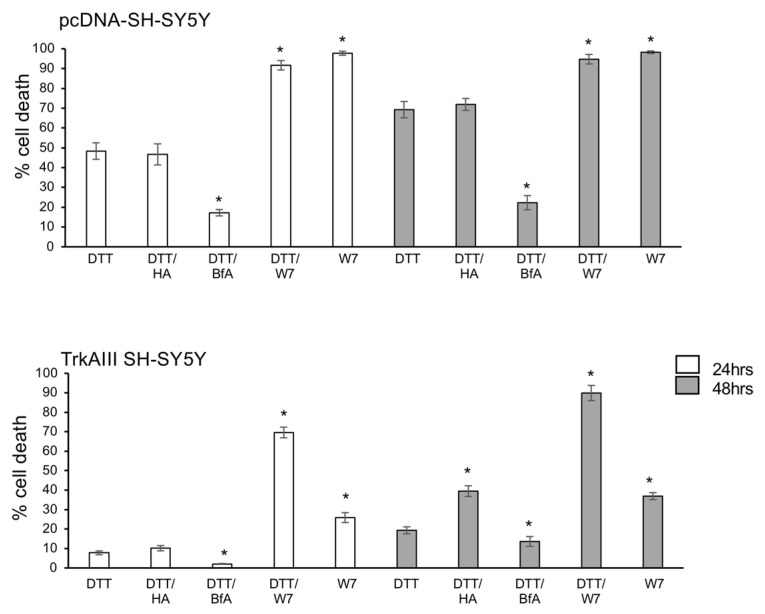
(**a**) Western blots demonstrating TrkAIII cleavage and phosphorylation in mitochondria (50 μg) from TrkAIII SH-SY5Y cells treated with 5 mM DTT alone for 6 h and in mitochondria from TrkAIII SH-SY5Y cells co-treated with 5 mM DTT and either HA-15 (20 μM) (DTT/HA); brefeldin A (5 mg/mL) (DTT/BfA) or W7 (60 μM) (DTT/W7) plus non-phosphorylated TrkAIII in mitochondria from untreated TrkAIII SH-SY5Y cells (Con) and lack of TrkA or phosphorylated TrkA immunoreactivity in mitochondria (50 μg) from untreated (Con) and DTT-treated (5 mm for 6 h) pcDNA-SH-SY5Y cells. (**b**) Line graphs demonstrating significant inhibition (*) of pcDNA-SH-SY5Y and TrkAIII SH-SY5Y proliferation by BfA (5 μg/mL) and W7 (60 μM) but not by HA-15 (20 μM) at 24 and 48 h (* *p* < 0.0001). (**c**) Phase contrast images merged with green fluorescence plus histograms demonstrating percentage pcDNA-SH-SY5Y and TrkAIII SH-SY5Y cell death induced by 5 mM DTT alone (DTT), DTT and either HA-15 (20 μM) (DTT/HA), BfA (5 μg/mL) (DTT/BfA) or W7 (60 μM) (DTT/W7), plus pcDNA-SH-SY5Y and TrkAIII SH-SY5Y cell death induced by W7 (60 μM) alone (W7) at 24 and 48 h (* *p* < 0.006).

In Western blots, mitochondrial TrkAIII cleavage and phosphorylation in TrkAIII SH-SY5Y cells induced by DTT treatment alone was not detected in mitochondrial extracts from TrkAIII SH-SY5Y co-treated with either DTT plus HA-15, DTT plus W7 or DTT plus BfA (Figure 3a).

As pcDNA-SH-SY5Y cells do not express TrkAIII protein [6] and TrkAIII was not detected in either untreated or DTT-treated pcDNA-SH-SY5Y mitochondria (Figure 3a), parallel experiments in pcDNA-SH-SY5Y cells were not performed.

Together, these data confirm the active participation of GRP78, Ca^2+^-calmodulin and Arf-1 in mitochondrial TrkAIII importation and cleavage and activation.

These inhibitors were also investigated on pcDNA-SH-SY5Y and TrkAIII SH-SY5Y proliferation and sensitivity to DTT-induced death. In duplicate proliferation assays repeated twice, mean (±s.d.) fold increase in pcDNA-SH-SY5Y or TrkAIII SH-SY5Y proliferation was not influenced by HA-15 alone over 48 h, whereas BfA completely blocked both pcDNA-SH-SY5Y and TrkAIII SH-SY5Y proliferation over 48 h but did not induce significant cell death, assessed by nuclear incorporation of IncuCyte Green Dye (Figure 3b). In contrast, W7 inhibited both pcDNA-SH-SY5Y and TrkAIII SH-SY5Y proliferation in association with significant cytotoxicity. In pcDNA-SH-SY5Y cells W7 induced 97.7 (±1)% cell death at 24 h and 98.25 (±0.64)% cell death at 48 h, and in TrkAIII SH-SY5Y cells, induced 25.9 (±2.5)% cell death at 24 h and 36.9 (±1.8)% cell death at 48 h. Levels of TrkAIII SH-SY5Y cell death induced by W7 were significantly lower at both 24 and 48 h than observed in pcDNA-SH-SY5Y cells (*p* < 0.0001 when compared to W7-induced pcDNA-SH-SY5Y cell death at 24 or 48 h, respectively) (Figure 3b,c).

Overall, these data implicate Arf1 but not Grp78 or Ca^2+^ calmodulin in SH-SY5Y and TrkAIII SH-SY5Y proliferation, which is in line with the essential role of Arf1 in cellular proliferation [47], and confirm that W7 disruption of Ca^2+^-calmodulin is cytotoxic to both pcDNA-SH-SY5Y and TrkAIII SH-SY5Y cells, in line with reports that W7 disruption of Ca^2+^ homeostasis induces apoptosis [45,48]. The significantly enhanced resistance of TrkAIII SH-SY5Y cells to W7-induced death compared to pcDNA-SH-SY5Y cells occurred in the absence of mitochondrial TrkAIII activation and, although not directly addressed in this study, is consistent with the enhanced constitutive expression of the Bcl2, Bcl-xL and Mcl-1 apoptosis inhibitor that characterizes TrkAIII SH-SY5Y cells [7,20].

In duplicate IncuCyte cytotoxicity assays, repeated at least twice, mean (±s.d.) pcDNA-SH-SY5Y cell death induced by DTT alone at 24 and 48 h was not significantly altered in cells co-treated with DTT and HA-15 (Figure 3c). In contrast, TrkAIII SH-SY5Y cell death induced by DTT alone was significantly increased at 48 h in cells co-treated with DTT plus HA-15 to 39.5 (±2.8)% (*p* < 0.0001 compared to TrkAIII SH-SY5Y cell treated for 48 h with DTT alone) and was also significantly increased in TrkAIII SH-SY5Y cells co-treated with DTT plus W7 to 69.6 (±2.6)% at 24 h (*p* < 0.0001 compared to TrkAIII SH-SY5Y cell treated for 24 h with DTT alone) and 89.9 (±3.9)% at 48 h (*p* < 0.0001 compared to TrkAIII SH-SY5Y cell treated for 48 h with DTT alone) (Figure 3c).

These data identify Grp78 and Ca^2+^-calmodulin as targetable participants in DTT-induced mitochondrial TrkAIII activation, the inhibition of which enhances sensitivity to DTT-induced death in TrkAIII SH-SY5Y cells. These data also demonstrate that DTT combined with W7 induces the highest levels of TrkAIII SH-SY5Y cell death, indicating that stress, combined with TrkAIII inhibition and deregulated Ca^2+^ homeostasis, is highly cytotoxic to TrkAIII-expressing NB cells.

In contrast to HA15 and W7, pcDNA-SH-SY5Y cell death induced by DTT alone was significantly reduced in cells co-treated with DTT plus BfA at 24 h to 17.2 (±1.6)% and at 48 h to 22.3 (±3.6)% (*p* < 0.0001 for both when compared to pcDNA-SH-SY5Y cells treated with DTT alone at each time point, respectively) and was also significantly reduced in TrkAIII SH-SY5Y cells co-treated with DTT plus BfA to 2.1 (±0.2)% at 24 h (*p* < 0.0001 compared to TrkAIII SH-SY5Y cells treated with DTT alone for 24 h) and 13.6 (±2.5)% at 48 h (*p =* 0.006 compared to TrkAIII SH-SY5Y cells treated with DTT alone for 48 h) (Figure 3c).

Together, these data confirm a direct role for Arf1 in DTT-induced death, despite involvement in DTT-induced mitochondrial TrkAIII activation.

Overall, these data identify Grp78 and Ca^2+^-calmodulin as novel targetable participants in DTT-induced mitochondrial TrkAIII cleavage and activation and in resistance to DTT-induced TrkAIII SH-SY5Y cell death and characterize Arf1 as a non-targetable participant DTT-induced mitochondrial TrkAIII cleavage and activation due to its direct involvement in DTT-induced death.

### 2.4. DTT-Induced Mitochondrial TrkAIII Activation Depends upon Hsp90, the MCU and ROS

Cytoplasmic TrkAIII activation depends upon Hsp90 [49], and DTT-induced mitochondrial TrkAIII activation requires intra-mitochondrial TrkAIII cleavage by the IMS serine protease Omi/HtrA2 [20,50]. Furthermore, mitochondrial Ca^2+^ uptake during integrated stress responses can activate the mitochondrial uniporter (MCU) [51], influencing metabolic ROS production [52,53]. We, therefore, investigated whether Hsp90, its mitochondrial analogue TRAP-1 [54], the MCU and ROS may also be required for DTT-induced mitochondrial TrkAIII importation, cleavage and activation.

**Figure 4:**
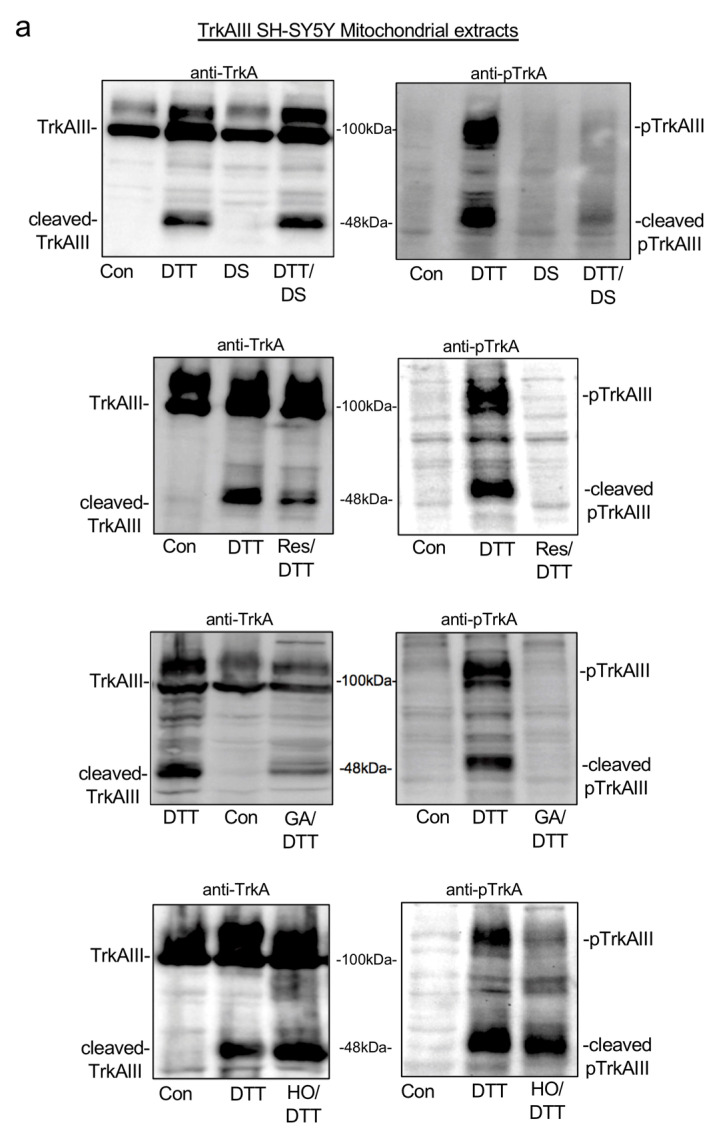

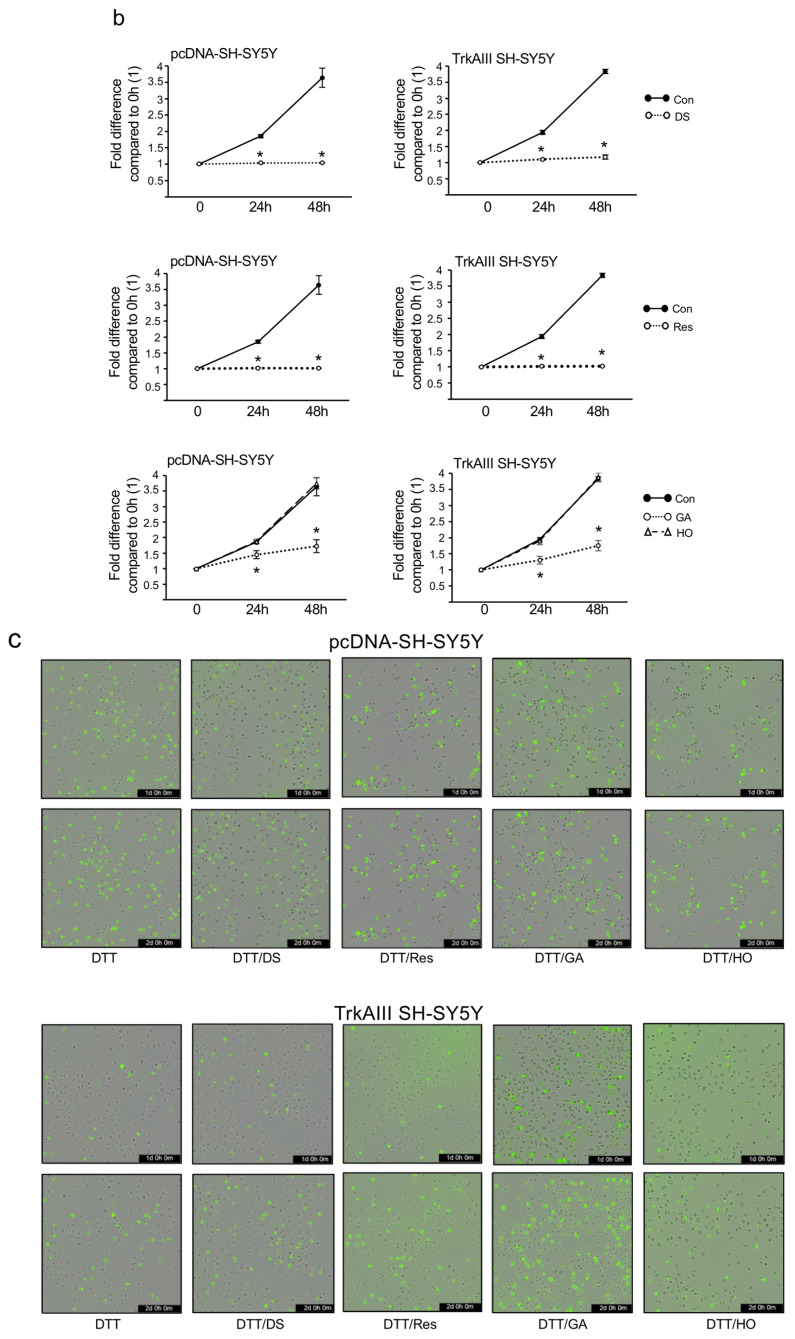

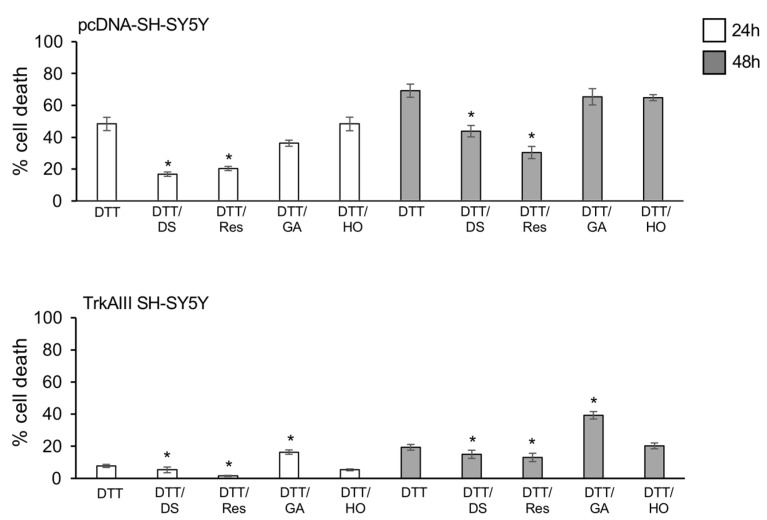
(**a**) Western blots of TrkAIII cleavage and phosphorylation in mitochondria (50 mg) from TrkAIII SH-SY5Y treated with 5 mM DTT for 6 h (DTT); co-treated with 5 mM DTT and either DS16570511 (40 μM) (DTT DS), resveratrol (100 μM) (DTT Res), geldanamycin (100 μM) (GA DTT) or Honokiol-DCA (10 μM) (DTT HON), plus un-cleaved, non-phosphorylated TrkAIII in mitochondria from untreated TrkAIII SH-SY5Y cells (Con). (**b**) Line graphs demonstrating the effects of DS16570511 (DS) (40 μM), resveratrol (Res) (100 μM), geldanamycin (GA) (100 μM) and Honokiol-DCA (HO) (10 μM) on pcDNA-SH-SY5Y and TrkAIII SH-SY5Y proliferation over 48 h (* *p* < 0.001). (**c**) Representative phase contrast images merged with green fluorescence and histograms comparing pcDNA-SH-SY5Y and TrkAIII SH-SY5Y cell death induced by 5 mM DTT to cell death induced by co-treatment with 5 mM DTT and either DS16570511 (40 μM) (DTT DS), resveratrol (100 μM) (DTT Res), geldanamycin (100 μM) (DTT GA) or Honokiol-DCA (10 μM) (DTT HO) (* *p <* 0.01).

In Western blots, compared to TrkAIII cleavage and phosphorylation in mitochondria from TrkAIII SH-SY5Y cells treated with DTT alone, TrkAIII phosphorylation but not cleavage was prevented in mitochondria from TrkAIII SH-SY5Y cells co-treated with DTT plus the mitochondrial uniporter (MCU) inhibitor DS1657051 [55] (Figure 4a). This implicates the MCU in DTT-induced phosphorylation of cleaved TrkAIII but not in TrkAIII cleavage.

In mitochondria from TrkAIII SH-SY5Y cells co-treated with either DTT plus the ROS scavenger resveratrol [56] or with DTT plus the Hsp90 inhibitor geldanamycin (GA) [57], TrkAIII phosphorylation was completely prevented, and cleavage was partially prevented (Figure 4a). These data implicate both ROS and Hsp90 in DTT-induced mitochondrial TrkAIII cleavage and phosphorylation. In contrast, neither the phosphorylation of cleaved mitochondrial TrkAIII nor TrkAIII cleavage were reduced in TrkAIII SH-SY5Y cells co-treated with DTT and the TRAP1 inhibitor Honokiol-DCA [58] (Figure 4a).

As pcDNA-SH-SY5Y cells do not express TrkAIII protein [6] and TrkAIII was not detected in either untreated or DTT-treated pcDNA-SH-SY5Y mitochondria (Figure 3a), parallel experiments in pcDNA-SH-SY5Y cells were not performed.

Overall, these data implicate Hsp90 and ROS in DTT-induced mitochondrial TrkAIII cleavage and phosphorylation and implicate the MCU in DTT-induced phosphorylation of cleaved TrkAIII but exclude TRAP-1 involvement in this mechanism.

These inhibitors were also tested on pcDNA-SH-SY5Y and TrkAIII SH-SY5Y proliferation and sensitivity to DTT-induced death. In duplicate proliferation assays, repeated three times, DS16570511 and resveratrol completely prevented pcDNA-SH-SY5Y and TrkAIII SH-SY5Y proliferation over 48 h, in line with the requirement for both the MCU and ROS for mitotic completion [53]. Geldanamycin also significantly inhibited pcDNA-SH-SY5Y and TrkAIII SH-SY5Y proliferation over 48 h, whereas Honokiol-DCA had no effect on either pcDNA-SH-SY5Y or TrkAIII SH-SY5Y proliferation (Figure 4b).

These data confirm requirements for the MCU and Hsp90 but not TRAP-1 in pcDNA-SH-SY5Y and TrkAIII SH-SY5Y proliferation.

Treatment with DS1657051, resveratrol and GA alone did not induce significant pcDNA-SH-SY5Y and TrkAIII SH-SY5Y cell death, assessed by nuclear IncuCyte Green Dye uptake.

In duplicate IncuCyte cytotoxicity assays, repeated a minimum of twice, mean (±s.d.) pcDNA-SH-SY5Y cell death induced by DTT alone was significantly reduced in cells co-treated with DTT plus DS16570511 to 16.82 (±1.37)% at 24 h and 43.8 (±3.59)% at 48 h (*p <* 0.0001 when compared to pcDNA-SH-SY5Y cells treated with DTT alone at 24 and 48 h, respectively). In TrkAIII SH-SY5Y cells, cell death induced by DTT alone was also significantly reduced in cells co-treated with DTT plus DS16570511 to 5.3 (±1.78)% at 24 h (*p =* 0.01 compared to TrkAIII cells treated with DTT alone at 24 h) and 15 (±2.53)% at 48 h (*p =* 0.006 compared to TrkAIII cells treated with DTT alone at 48 h) (Figure 4c). These data directly implicate the MCU directly in DTT-induced pcDNA-SH-SY5Y and TrkAIII SH-SY5Y cell death.

PcDNA-SH-SY5Y cell death induced by DTT alone was also significantly reduced in cells co-treated with DTT plus resveratrol to 20.4 (±1.3)% at 24 h and 30.4 (±3.8%) at 48 h (*p* < 0.0001 when compared to pcDNA-SH-SY5Y cells treated with DTT alone for 24 h and 48 h, respectively). TrkAIII SH-SY5Y cell death induced by DTT alone was also significantly reduced in cells co-treated with DTT and resveratrol to 1.5 (±0.4)% cell death at 24 h (*p =* 0.01 compared to death induced by DTT alone at 24 h) and 13.05 (±2.6)% at 48 h (*p =* 0.006 compared to TrkAIII SH-SY5Y cell death induced by DTT alone at 48 h) (Figure 4c). These data implicate ROS directly in DTT-induced pcDNA-SH-SY5Y and TrkAIII SH-SY5Y cell death.

PcDNA-SH-SY5Y cell death induced by DTT alone was not significantly altered in cells co-treated with DTT plus GA at either 24 or 48 h (Figure 4b) but was significantly increased in TrkAIII SH-SY5Y cells co-treated with DTT and GA to 16.4 (±1.4)% at 24 h and 39.3 (±2.3)% at 48 h (*p* < 0.0001 when compared to TrkAIII SH-SY5Y cells treated with DTT alone for 24 h and 48 h, respectively). In contrast, co-treatment of pcDNA-SH-SY5Y and TrkAIII SH-SY5Y cells with DTT plus Honokiol-DCA had no significant effect on DTT-induced cell death (Figure 4c).

These data confirm a role for Hsp90 in TrkAIII SH-SY5Y but not pcDNA-SH-SY5Y in resistance to DTT-induced death and exclude TRAP-1 involvement.

Overall, these data identify Hsp90 as a targetable participant in DTT-induced mitochondrial TrkAIII activation, the inhibition of which enhanced DTT-induced TrkAIII SH-SY5Y cell death, and identify the MCU and ROS as not targetable participants in DTT-induced mitochondrial TrkAIII activation due to direct involvement in DTT-induced cell death.

### 2.5. DTT-Induced Mitochondrial TrkAIII Activation Depends upon CAMKII and Akt

Clinically approved Entrectinib [59] and Lestaurtinib [60] Trk inhibitors were also used to evaluate whether TrkAIII activation was required for DTT-induced mitochondrial TrkAIII importation and cleavage.

**Figure 5:**
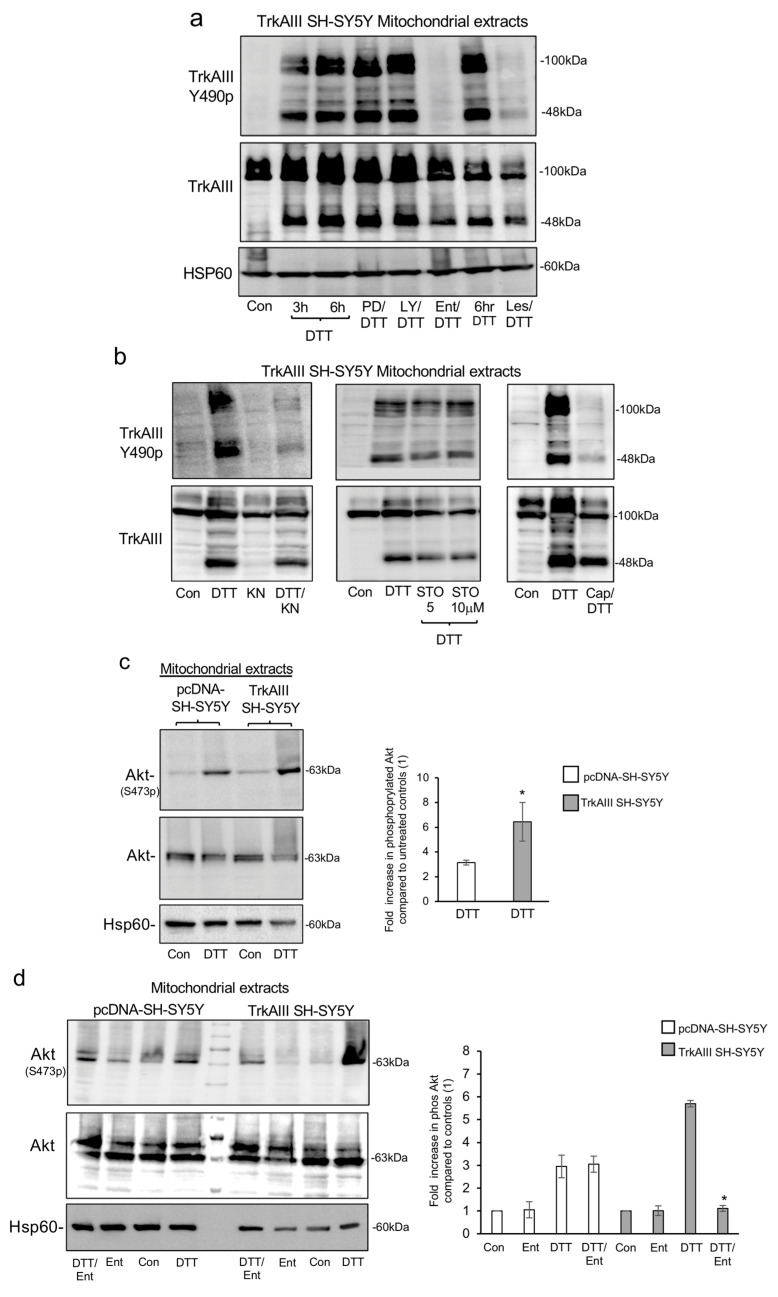

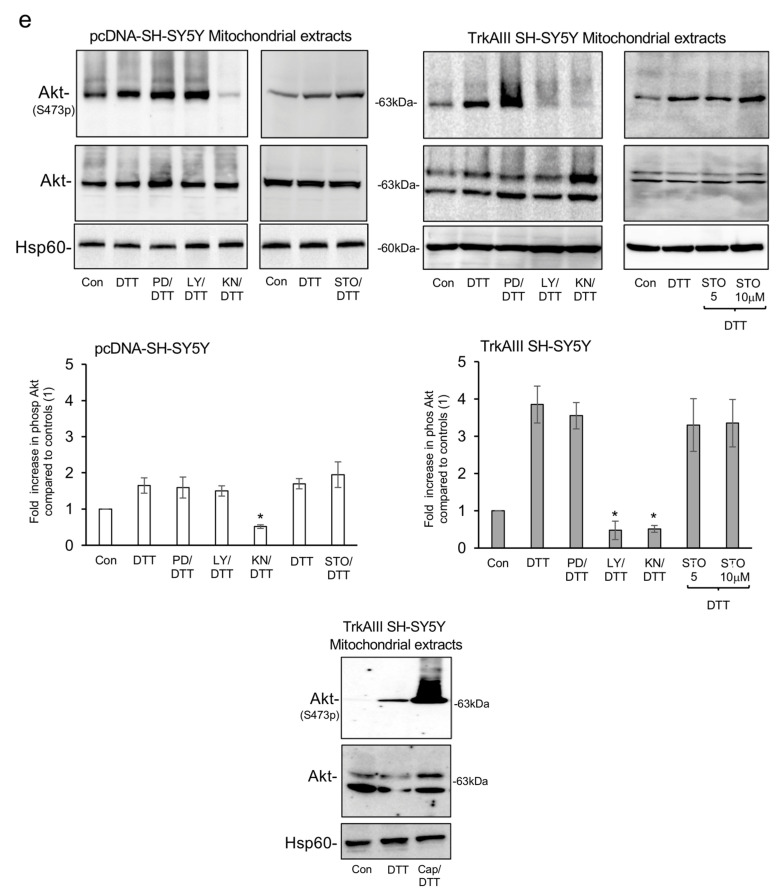

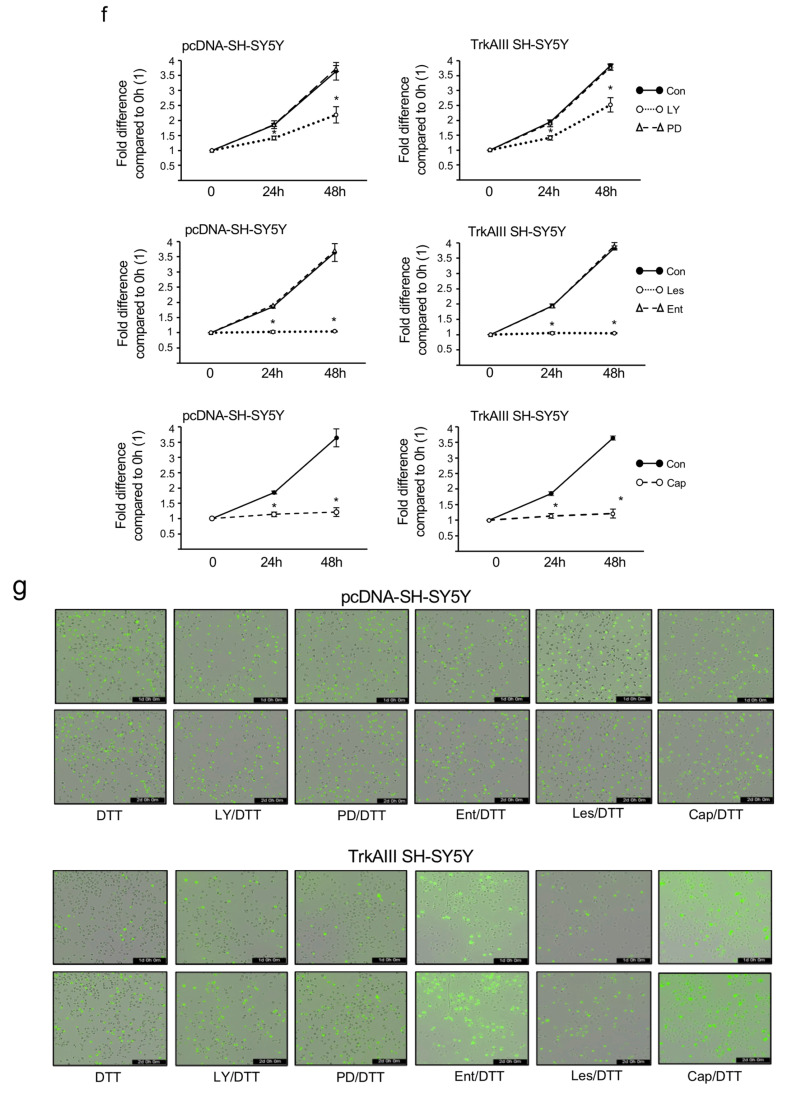

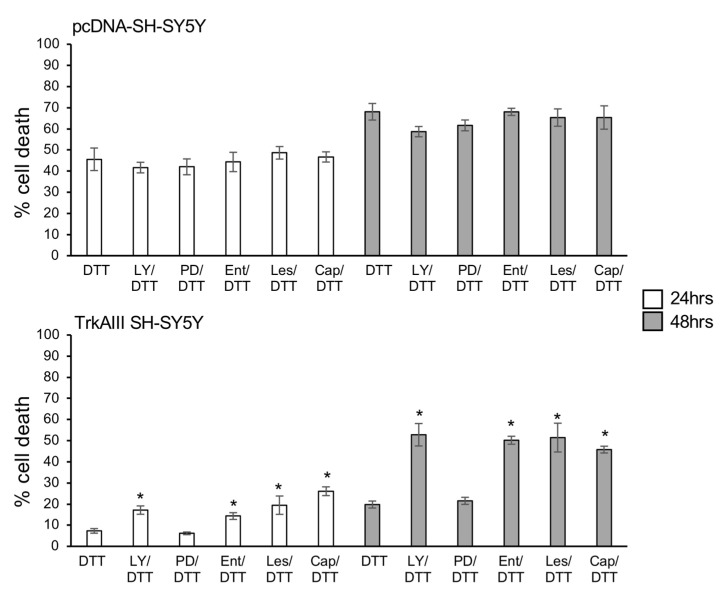
(**a**) Western blots of TrkAIII cleavage and phosphorylation in mitochondria (50 μg) from TrkAIII SH-SY5Y cells treated for 3 and 6 h with 5 mM DTT and from TrkAIII SH-SY5Y cells co-treated for 6 h with 5 mM DTT and either PD098059 (10 μM) (DTT/PD), LY294002 (25 μM) (DTT/LY), Entrectinib (1 μM) (DTT/Ent) or Lestaurtinib (1 μM) (DTT/Les). (**b**) Western blots of TrkAIII cleavage and phosphorylation in mitochondria (50 μg) from TrkAIII SH-SY5Y cells treated for 6 h with 5 mM DTT and from TrkAIII SH-SY5Y cells co-treated for 6 h with 5 mM DTT plus either KN-93 (10 μM) (DTT/KN), STO-609 (5 μM and 10 μM) (STO/DTT) or Capivasertib (20 μM) (DTT/Cap) plus un-cleaved non-phosphorylated TrkAIII in mitochondria from untreated TrkAIII SH-SY5Y cells (Con). (**c**) Western blots demonstrating Ser473 phosphorylated and total Akt in mitochondria (50 μg) from pcDNA-SH-SY5Y and TrkAIII SH-SY5Y cells treated for 6 h with 5 mM DTT compared to untreated controls (Con) and histogram comparing mean (±s.d.) fold increase in mitochondrial Akt S473 phosphorylation compared to untreated controls (* *p* < 0.0221); (**d**) Western blots demonstrating Ser473 phosphorylated and total Akt in mitochondria (50 μg) from pcDNA-SH-SY5Y and TrkAIII SH-SY5Y cells treated for 6 h with either 5 mM DTT alone (DTT), 1 μM Entrectinib alone (Ent) or co-treated for 6 h with 5 mM DTT plus Entrectinib (1 μM) (DTT Ent) compared to levels in mitochondria from untreated pcDNA-SH-SY5Y and TrkAIII SH-SY5Y controls (Con), and accompanying histogram and histogram comparing mean (±s.d.) fold increase in mitochondrial Akt S473 phosphorylation compared to untreated controls demonstrating significant inhibition of DTT-induced mitochondrial Akt S473 phosphorylation in TrkAIII SH-SY5Y but not pcDNA-SH-SY5Y cells co-treated with DTT and 1 μM Entrectinib inhibition (* *p* <0.0009 compared to Akt S473 phosphorylation levels in TrkAIII SH-SY5Y cells treated with DTT alone). (**e**) Western blots of Ser473 phosphorylated and non-phosphorylated Akt levels in mitochondria (50 μg) from pcDNA-SH-SY5Y and TrkAIII SH-SY5Y cells treated for 6 h with 5mM DTT alone (DTT) or counterparts co-treated for 6 h with 5 mM DTT and either PD098059 (10 μM) (DTT/PD), KN-93 (10 μM) (DTT/KN), LY294002 (25 μM) (DTT/LY) or STO-609 (5 μM and/or 10 μM) (DTT/STO), plus a histogram demonstrating significant inhibition of DTT-induced mean (±s.d.) fold increase in Akt S473 phosphorylation induced in pcDNA-SH-SY5Y cells, in cells co-treated with DTT-and KN-93 (* *p* = 0.03 compared to DTT alone) but not with LY294002, and significant reductions in DTT-induced mitochondrial Akt S473 phosphorylation in TrkAIII SH-SY5Y cells co-treated with DTT and either KN-93 (* *p* = 0.011) or LY294002 (* *p* = 0.013). The bottom panel demonstrates the increase in DTT-induced mitochondrial Akt S473 phosphorylation caused by Capivasertib (20 μM) in TrkAIII SH-SY5Y cells (DTT/Cap). (**f**) Line graphs demonstrating the effects of PD098059 (10 μM), LY294002 (25 μM) (LY), Entrectinib (1 μM), Lestaurtinib (1 μM) (Les) and Capivasertib (20 μM) (Cap) on pcDNA-SH-SY5Y and TrkAIII SH-SY5Y proliferation over 48 h (* *p* < 0.0001). (**g**) Representative phase contrast images merged with green fluorescence and histograms of mean (±s.d.) percentage of pcDNA-SH-SY5Y and TrkAIII SH-SY5Y cell death at 24 and 48 h, induced by 5 mM DTT alone (DTT) or by DTT co-treatment with either LY294002 (25 μM) (DTT/LY), PD098059 (10 μM) (DTT/PD), Entrectinib (1 μM) (DTT/Ent), Lestaurtinib (1 μM) (DTT/Les) or Capivasertib (20 μM) (DTT/Cap) (* *p* < 0.0001).

In Western blots, compared to TrkAIII cleavage and phosphorylation in TrkAIII SH-SY5Y mitochondria treated with DTT alone for 3 or 6 h, co-treatment of cells with DTT plus Entrectinib (Ent/DTT) or with DTT plus Lestaurtinib (Les/DTT) completely prevented DTT-induced mitochondrial TrkAIII phosphorylation but not cleavage (Figure 5a).

Together, these data confirm that Entrectinib and Lestaurtinib inhibit mitochondrial TrkAIII activation and that DTT-induced mitochondrial TrkAIII importation and cleavage are independent of TrkAIII activity.

Cytoplasmic TrkAIII activation induces PI3K/Akt signaling [6], PI3K/Akt activation regulates mitochondrial function and survival [61,62], and Akt can be activated by MAPK [63], PI3K [64] and CAMK-CAMKK pathways [65,66]. We, therefore, investigated the relationship between DTT-induced mitochondrial TrkAIII activation, MAPK, PI3K and CAMK pathways, and mitochondrial Akt S473 phosphorylation, using the MAPK inhibitor PD090859 [67], the PI3K inhibitor LY290042 [68], the CaMKI, II and IV inhibitor KN-93 [69], the CAMKKα/β, CaMKI and IV inhibitor STO-609 [70] and the Akt inhibitor Capivasertib (AZD5363) [71].

In Western blots, TrkAIII cleavage and phosphorylation in mitochondrial extracts from TrkAIII SH-SY5Y cells treated for 6 h with DTT alone were not altered in mitochondrial extracts from cells co-treated with DTT and PD090859 (PD/DTT) or LY290042 (LY/DTT) (Figure 5a), excluding PI3K and MAPK pathway involvement. In contrast, TrkAIII SH-SY5Y cells co-treated with DTT and KN-93 (KN/DTT) or Capivasertib (Cap/DTT) DTT-induced mitochondrial TrkAIII phosphorylation but not cleavage were prevented by both co-treatments, whereas co-treatment with DTT plus STO-609 (STO/DTT) had no effect (Figure 5b).

As pcDNA-SH-SY5Y cells do not express TrkAIII protein [6] and TrkAIII was not detected in either untreated or DTT-treated pcDNA-SH-SY5Y mitochondria (Figure 3a), parallel experiments in pcDNA-SH-SY5Y cells were not performed.

Together, these data unveil unexpected roles for CAMKII and Akt in DTT-induced mitochondrial TrkAIII phosphorylation but not cleavage and exclude CAMKK cascade involvement.

### 2.6. DTT-Induced Akt Ser473 Phosphorylation Depends upon CAMKII in pcDNA-SH-SY5Y Cells and CAMKII, TrkAIII and PI3K in TrkAIII SH-SY5Y Cells

The unexpected Akt requirement for DTT-induced mitochondrial TrkAIII phosphorylation and potential of mitochondrial TrkAIII to activate a pro-survival PI3K–Akt axis prompted further analysis of Akt involvement in this mechanism.

In duplicate densitometric Western blot assays, DTT treatment of both pcDNA-SH-SY5Y and TrkAIII SH-SY5Y cells induced mitochondrial Akt serine (S) 473 phosphorylation, consistent with full Akt activation [72], and in DTT-treated TrkAIII SH-SY5Y cells the mean (±s.d.) percentage fold increase in Akt S473 phosphorylation was significantly higher than in pcDNA-SH-SY5Y cells (*p =* 0.0221) (Figure 5c,d). These data indicate that DTT-induced mitochondrial Akt activation in pcDNA-SH-SY5Y cells is TrkAIII-independent but TrkAIII augments DTT-induced Akt activation in TrkAIII SH-SY5Y cells.

To confirm TrkAIII involvement in mitochondrial Akt activation, Akt S473 phosphorylation levels in mitochondria from untreated pcDNA-SH-SY5Y and TrkAIII SH-SY5Y cells (Con) were compared to Akt S473 phosphorylation levels in mitochondria from pcDNA- SH-SY5Y and TrkAIII SH-SY5Y cells treated with DTT alone (DTT), Entrectinib alone (Ent) or co-treated with DTT plus Entrectinib (Ent/DTT).

In duplicate densitometric Western blot assays, mitochondrial Akt S473 phosphorylation in pcDNA-SH-SY5Y treated with DTT alone was not reduced in mitochondria from cells co-treated with DTT plus Entrectinib (Figure 5d left, four panels and histogram). In contrast, mitochondrial Akt S473 phosphorylation in TrkAIII SH-SY5Y treated with DTT alone was significantly reduced in mitochondria from cells co-treated with DTT and Entrectinib (Figure 5d right, four lanes and histogram) (*p* < 0.0009 compared to TrkAIII SH-SY5Y cells treated DTT treatment alone). Treatment of pcDNA-SH-SY5Y or TrkAIII SH-SY5Y cells with Entrectinib alone did not alter mitochondrial Akt S473 phosphorylation. These data confirm that the DTT-induced increase in mitochondrial Akt S473 phosphorylation in TrkAIII SH-SY5Y cells but not in pcDNA-SH-SY5Y cells depends upon Entrectinib-inhibitable tyrosine kinase activity, implicating TrkAIII.

In additional experiments, mitochondrial Akt S473 phosphorylation in untreated pcDNA-SH-SY5Y and TrkAIII SH-SY5Y cells (Con) was compared to mitochondrial Akt S473 phosphorylation in pcDNA-SH-SY5Y and TrkAIII SH-SY5Y cells treated with DTT alone (DTT) or co-treated with DDT and either PD-090859 (PD/DTT), LY290042 (LY/DTT), KN-93 (KN/DTT), STO-609 (STO/DTT) or Capivasertib (Cap/DTT).

In duplicate densitometric Western blot assays, mitochondrial Akt S473 phosphorylation in pcDNA-SH-SY5Y cells treated with DTT alone was not significantly reduced in cells co-treated with DTT with either PD-090859 (PD/DTT), LY290042 (LY/DTT) or STO-609 (STO/DTT) but was significantly reduced in pcDNA-SH-SY5Y cells co-treated with DTT and KN-93 (KN/DTT) (*p* = 0.03 compared to pcDNA-SH-SY5Y cells treated with DTT alone) (Figure 5e left, two panels and histogram). In contrast, mitochondrial Akt S473 phosphorylation in TrkAIII SH-SY5Y cells treated with DTT alone was significantly reduced in TrkAIII SH-SY5Y cells co-treated with DTT and either LY290042 (LY/DTT) (* *p* = 0.013 compared to TrkAIII SH-SY5Y cells treated with DTT alone) or KN-93 (KN/DTT) (* *p* = 0.011 compared to TrkAIII SH-SY5Y cells treated with DTT alone) but was not significantly altered in cells co-treated with DTT and either PD-090859 or STO-609 (STO/DTT) (Figure 5e right, two panels and histogram).

These data implicate CAMKII and exclude CAMKKα/β, CaMKI, CaMKIV and the MAPK pathway in DTT-induced mitochondrial Akt S473 phosphorylation in both pcDNA-SH-SY5Y and TrkAIII SH-SY5Y cells and additionally implicate PI3K in DTT augmented mitochondrial Akt S473 phosphorylation in TrkAIII SH-SY5Y cells.

Of note, TrkAIII SH-SY5Y co-treatment with DTT and Capivasertib markedly augmented mitochondrial Akt S473 phosphorylation (Figure 5e, bottom panels), in line with a previous report [71].

### 2.7. TrkAIII SH-SY5Y Resistance to DTT-Induced Death Depends upon PI3K and Akt

To evaluate the influence of mitochondrial TrkAIII-dependent, PI3K-mediated Akt activation on resistance to DTT-induced death, pcDNA-SH-SY5Y and TrkAIII SH-SY5Y cells were compared in proliferation and IncuCyte cytotoxicity assays in the presence of DTT and PD-090859, LY290042, Entrectinib, Lestaurtinib and Capivasertib inhibitors.

In proliferation assays, Lestaurtinib and Capivasertib almost completely inhibited pcDNA-SH-SY5Y and TrkAIII SH-SY5Y proliferation over 48 h. LY294002 also significantly reduced pcDNA-SH-SY5Y and TrkAIII SH-SY5Y proliferation at both 24 and 48 h, whereas PD098059 and Entrectinib had no effect on either pcDNA-SH-SY5Y or TrkAIII SH-SY5Y proliferation over 48 h (Figure 5f).

These data implicate tyrosine kinases inhibited by Lestaurtinib but not by Entrectinib and also PI3k and Akt in pcDNA-SH-SY5Y and TrkAIII SH-SY5Y proliferation and also exclude a direct role for TrkAIII in TrkAIII SH-SY5Y proliferation. These inhibitors did not induce significant cell death alone, assessed by nuclear IncuCyte Green Dye uptake.

In IncuCyte cytotoxicity assays, the high levels of pcDNA-SH-SY5Y cell death induced by DTT alone at 24 and 48 h were not significantly increased in pcDNA-SH-SY5Y cells co-treated with DTT and either LY294002, PD098059, Entrectinib, Lestaurtinib or Capivasertib (Figure 5g, upper panels and upper histogram). In contrast, lower levels of DTT-induced TrkAIII SH-SY5Y cell death were significantly increased in cells co-treated with DTT and LY294002 (mean ± s.d.) to 14.5 (±0.5) % at 24 h and 43.4 (±3.8)% at 48 h (*p* < 0.001 compared to TrkAIII SH-SY5Y cells treated with DTT at 24 h and 48 h, respectively); DTT and Entrectinib to 14.3 (±1.6)% at 24 h and 50.2 (±1.9)% at 48 h (*p* < 0.001 compared to TrkAIII SH-SY5Y cells treated with DTT at 24 h and 48 h, respectively); DTT and Lestaurtinib to 19.5 (±4.3)% at 24 h and 51.4 (±6.9)% at 48 h (*p* < 0.001 compared to TrkAIII SH-SY5Y cells treated with DTT at 24 h and 48 h, respectively) or DTT and Capivasertib to 26.1 (±2.1)% at 24 h and 45.8 (±1.6)% at 48 h (*p* < 0.001 compared to TrkAIII SH-SY5Y cells treated with DTT at 24 h and 48 h, respectively), but they were not significantly altered by DTT co-treatment with PD098059 (Figure 5g, lower panels and lower histogram).

These data implicate PI3K and Akt but not the MAPK pathway in the resistance of TrkAIII SH-SY5Y cells to DTT-induced death and confirm that clinically approved Trk (Entrectinib and Lestaurtinib) and Akt (Capivasertib) inhibitors and PI3K inhibitor LY294002 significantly increase the sensitivity of TrkAIII-expressing pcDNA-SH-SY5Y cells to DTT-induced death.

The effects of all of the inhibitors used in this study are summarized in Table 1.

## 3. Discussion

**Figure 6:**
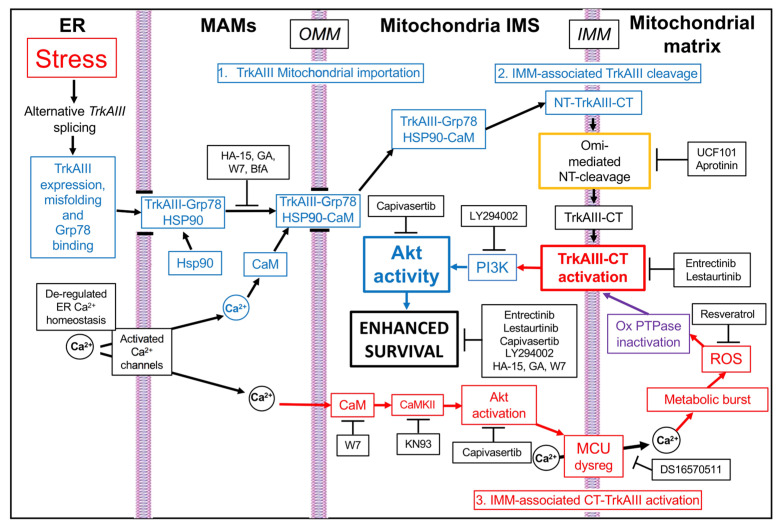
Schematic representation of the pro-survival mechanism investigated in this study, including the following: 1. Grp78, Hsp90, Ca^2+^-calmodulin and ARF1 regulated misfolded TrkAIII importation from the endoplasmic reticulum (ER) into the inner mitochondrial membrane (IMM) via MAMs; 2. Omi cleavage of the IMM-associated misfolded TrkAIII N-terminus, required for subsequent activation of the IMM-associated TrkAIII C-terminus; and 3. Ca^2+^-calmodulin- (CaM), CaMK II-, Akt-, MCU- and ROS-dependent activation of the cleaved IMM-associated TrkAIII C-terminus, associated with inhibitory mitochondrial PTPase oxidation. The inhibitors used in this study are localized at their points of action, and the inhibitors that reduce TrkAIII SH-SY5Y resistance to DTT-induced death are indicated by the side of “enhanced survival”.

In this investigation of molecular participants in a novel stress-induced pro-survival adaptation of the integrated stress response in TrkAIII-expressing SH-SY5Y NB cells, we characterize misfolding as a prerequisite for stress-induced mitochondrial importation and cleavage activation of the TrkAIII oncoprotein. We identify GRP-78, Ca^2+^-calmodulin, Hsp90, and Akt, in addition to TrkAIII, as novel targetable participants in this mechanism, the inhibition of which enhances TrkAIII-expressing SH-SY5Y sensitivity to DTT-induced death, and characterize Arf-1, the MCU and ROS as non-targetable participants in this mechanism due to their direct involvement in DTT-induced death. In this mechanism (Figure 6), stress induces TrkAIII misfolding and complexing with GRP78, which combined with increased TrkAIII complexing with Ca^2+^-calmodulin, results in GRP78, Ca^2+^-calmodulin, Hsp90 and Arf1-regulated misfolded TrkAIII importation into mitochondrial IMMs. IMM-associated misfolded TrkAIII N-termini are then degraded by Omi/HtrA2 [13], resulting in the accumulation of IMM-associated correctly folded TrkAIII C-termini, which are subsequently activated by a CAMKII-, Akt-, MCU- and ROS-dependent mechanism, in association with inhibitory oxidation of mitochondrial PTPases. Intra-mitochondrial TrkAIII activation augments mitochondrial Akt activation via PI3K, enhancing survival as part of a stress-regulated pro-survival mitochondrial TrkAIII–PI3K–Akt axis. Prevention of this mechanism by Trk (Entrectinib and Lestaurtinib), Akt (Capivasertib), GRP78 (HA-15) [31], Ca^2+^-calmodulin (W7) [32], Hsp90 (GA) [36], Arf1 (BfA) [33], MCU (DS1657051) [34] and ROS (resveratrol) [35] inhibitors confirms participation. Of these inhibitors, HA-15, W7, GA, Capivasertib, Entrectinib and Lestaurtinib significantly increase the sensitivity of TrkAIII-expressing SH-SY5Y cells to DTT-induced death, identifying Grp78, Ca^2+^-calmodulin, Hsp90 and Akt as novel targetable participants, in addition to TrkAIII. The LY294002 PI3K inhibitor [43] also enhanced TrkAIII SH-SY5Y sensitivity to DTT-induced death but did not prevent mitochondrial TrkAIII activation, confirming PI3K involvement downstream of mitochondrial TrkAIII activation. In contrast, BfA, DS1657051 and resveratrol all significantly enhanced TrkAIII SH-SY5Y resistance to DTT-induced death, confirming that Arf1, the MCU and ROS directly participate in DTT-induced death and are, therefore, unsuitable targets in this mechanism.

DTT was chosen as an activator of this mechanism for further investigation of the molecular requirements and participants in mitochondrial TrkAIII importation and cleavage activation. This agent recapitulates several stresses (ER, Ca^2+^, redox and hypoxic stress) experienced by tumor cells within the tumor microenvironment and activates integrated stress responses in cell models, including SH-SY5Y NB cells (this study) and [7,19,20,41,73,74,75]. The requirement for TrkAIII misfolding for stress-induced mitochondrial importation and cleavage activation was confirmed by increased TrkAIII complexing with the misfolded protein-binding ER chaperone Grp78 [39] and also by the cleavage of IMM-associated TrkAIII N-termini by the serine protease OMI/HtrA2, which degrades misfolded proteins within the IMS [31]. This is consistent with the presence of redox-sensitive cysteine-rich domains in the TrkAIII N-terminus [76]. DTT also caused the loss of TrkAIII immunoreactivity in non-reducing but not reducing SDS-PAGE Western blots, indicating reduction-sensitive masking of the TrkAIII distal C-terminus epitope recognized by the anti-TrkA antibody used in this study. This can be explained by the increase in complexing between TrkAIII and Ca^2+^-calmodulin induced by DTT, which extends a previous report that Ca^2+^-calmodulin binds the fully spliced TrkA distal C-terminus [40] to include TrkAIII, which also contains this domain [6].

HA-15, W7, GA and BfA all prevented DTT-induced mitochondrial TrkAIII cleavage and activation, confirming roles for GRP78, Ca^2+^-calmodulin, Hsp90 and Arf1, respectively, in DTT-induced mitochondrial misfolded TrkAIII importation and cleavage activation. This in line with reports that state that (i) agents that cause mitochondrial TrkAIII cleavage activation [20] also promote GRP78 re-localization from the ER to the inner mitochondrial membrane space [77]; (ii) RyR Ca^2+^ channels expressed by SH-SY5Y cells [78], are activated by DTT [73] and inhibited by dantrolene, which blocks DTT-induced mitochondrial TrkAIII activation, implicating Ca^2+^ in TrkAIII movement from the ER into mitochondria [20]; (iii) Hsp90 regulates mitochondrial protein delivery [79], is required for TrkAIII activation [49], and accumulates within mitochondria during stressful conditions [80]; and (iv) TrkAIII localizes to MAMs [20], MAM formation and function is regulated by Arf1 [81], and MAMs are involved in stress-induced mitochondrial importation of misfolded proteins and Ca^2+^ [24,42,43]. In contrast, the mitochondrial Hsp90 analogue TRAP1 inhibitor Honokiol-DCA [58,82] did not prevent DTT-induced mitochondrial TrkAIII cleavage activation, excluding TRAP1 involvement, supported by reports that TrkAIII is an HSP90 client [49] and TRAP1 does not bind Hsp90 clients [54]. HA-15, W7 and GA also significantly increased TrkAIII SH-SY5Y sensitivity to DTT-induced death, identifying Grp78, Ca^2+^-calmodulin and Hsp90 as novel targets in this pro-survival adaptation. In contrast, and despite preventing mitochondrial TrkAIII activation, BfA enhanced pcDNA-SH-SY5Y and TrkAIII SH-SY5Y resistance to DTT-induced death, identifying Arf1 as a direct participant in DTT-induced death and, therefore, as an unsuitable target.

Ca^2+^-calmodulin involvement in mitochondrial misfolded TrkAIII importation is reminiscent of its role in mitochondrial importation of tryparedoxin peroxidase in the unicellular eukaryote *Leishmania donovani* and may represent the first example of a similar Ca^2+^-calmodulin-dependent mitochondrial protein translocation mechanism in multicellular eukaryotes [83]. Furthermore, intra-mitochondrial TrkAIII activation was dependent upon Ca^2+^-calmodulin, suggesting that misfolded TrkAIII may act as a novel stress-regulated Ca^2+^-calmodulin-dependent mitochondrial tyrosine kinase. TrkAIII, however, does not possess classical mitochondrial targeting sequences and is not internalized into mitochondria under normal conditions [20], identifying GRP78, Ca^2+^-calmodulin and/or Hsp90 binding sites within misfolded TrkAIII as effective stress-regulated mitochondrial TrkAIII importation signals. W7 was also unique in eliciting high-level pcDNA-SH-SY5Y cell death alone, consistent with reports that calmodulin antagonists, including W7, alter calmodulin-dependent mitochondrial Ca^2+^ homeostasis, trigger mptp opening and cause mitochondrial death [45,48]. TrkAIII SH-SY5Y cells, however, were significantly more resistant to W7 cytotoxicity. This was not associated with mitochondrial TrkAIII activation, and we assume it relates to the elevated Bcl-xL, Mcl1 and SOD2 expression that characterize the TrkAIII SH-SY5Y cell line [7,20].

Within mitochondria, misfolded IMM-associated TrkAIII is cleaved by the IMS serine protease Omi/HtrA2 [26,30,31,50,84] to a 48 kDa C-terminus fragment [20]. This fragment contains TrkAIII tyrosine kinase and transmembrane domains and is separated from the N-terminus by the IMM [20]. Omi/HtrA2, combined with other mitochondrial protein quality control and proteostasis IMS, IMM and matrix proteases, degrades misfolded proteins that enter mitochondria under condition of stress [26,30,31,32,33,50,84]. Omi/HtrA2 degradation of IMM-associated TrkAIII N- but not C-termini, therefore, confirms misfolding of the former but not the latter and also suggests a predominant mitochondrial matrix orientation for IMM-associated TrkAIII C-termini. Dependence for mitochondrial TrkAIII activation upon cleavage, furthermore, supports an activation-preventing function for the misfolded TrkAIII N-terminus within mitochondria, analogous to a similar function for the fully spliced TrkA N-terminus at the cell surface [9,10]. Following cleavage, however, activated TrkAIII C-termini appear to trans-activate uncleaved TrkAIII within mitochondria, further augmenting mitochondrial TrkAIII activation levels (this study) and [20]. This highlights the critical role for Omi/HhtrA2 in this activation process, which could extend to the cleavage activation of other misfolded oncogenic receptor tyrosine kinases that re-localize to mitochondrial IMMs under conditions of stress [85].

KN-93 [69], Capivasertib [71] and DS1657051 [55] all prevented DTT-induced mitochondrial TrkAIII phosphorylation but not cleavage, verifying roles for CaMK(s), Akt and the MCU, respectively, in the activation of cleaved mitochondrial TrkAIII C-termini. The ROS scavenger resveratrol [56] also prevented DTT-induced mitochondrial TrkAIII phosphorylation and partially inhibited TrkAIII cleavage, implicating ROS in both mitochondrial TrkAIII cleavage and activation. The unexpected Akt requirement for TrkAIII activation was also supported by the KN-93 prevention of Akt serine 473 phosphorylation in pcDNA-SH-SY5Y and TrkAIII SH-SY5Y cells, linking mitochondrial CAMK(s) to mitochondrial Akt and subsequent TrkAIII activation. Akt, however, is a serine/threonine kinase, indicating an indirect role in TrkAIII tyrosine kinase activation. Considering the MCU and ROS involvement in mitochondrial TrkAIII activation, this role may depend upon Akt phosphorylation of the MCU regulatory subunit MICU1 [86], reported to deregulate MCU function, increase Ca^2+^ influx into the mitochondrial matrix and enhance ROS production [52,86,87,88,89,90,91,92,93,94,95,96,97,98]. Furthermore, reports that PTPase inhibitors promote TrkAIII activation [19] and H_2_O_2_ activates OMM-associated TrkAIII in mitochondria from non-stressed TrkAIII SH-SY5Y cells [20], combined with the increase in mitochondrial PTPases oxidation induced by DTT in this study, implicate oxidative inhibition of mitochondrial PTPases [38,89] in explanation for ROS involvement in mitochondrial TrkAIII activation.

DTT induced significantly higher levels of mitochondrial Akt serine 473 phosphorylation in TrkAIII SH-SY5Y compared to pcDNA-SH-SY5Y cells. Furthermore, KN-93, an inhibitor of CaMKI, II and IV [69,90], prevented Akt phosphorylation in pcDNA-SH-SY5Y and TrkAIII SH-SY5Y cells, whereas Trk (Entrectinib) [59] and PI3K (LY29004) [68] inhibitors prevented DTT-induced Akt phosphorylation only in TrkAIII SH-SY5Y cells. In contrast to KN-93, STO-609, an inhibitor CaMKKα/β that also prevents CaMKI and IV but not CaMKII activation [70,90], did not prevent DTT-induced Akt phosphorylation in either pcDNA-SH-SY5Y or TrkAIII SH-SY5Y cells, implicating CaMKII, rather than a CaMKK cascade, in DTT-induced Akt activation, in line with reports that CAMKII activates Akt [91,92]. This suggests that initial DTT-induced CaMKII-dependent phosphorylation of Akt S473 is followed by Akt-dependent mitochondrial cleaved TrkAIII activation, which further augments Akt S473 phosphorylation via PIP3K, also in line with previous reports [6,93,94].

Trk (Entrectinib and Lestaurtinib) [59,60], PI3K (LY294002) [68] and Akt (Capivasertib) [71] inhibitors all significantly enhanced TrkAIII SH-SY5Y sensitivity to DTT-induced death, confirming a pro-survival function for the stress-induced mitochondrial TrkAIII–PI3K–Akt axis, identifying mitochondrial PI3K and Akt as novel targets. This is consistent with reports that TrkAIII induces PI3K/Akt signaling and enhances mitochondrial Bcl2, Bcl-xL and Mcl1 expression in SH-SY5Y cells [6,20], Mcl-1 prevents mptp opening [95] and Akt enhances the anti-apoptotic activity of Bcl2, Bcl-xL and Mcl1 [96,97,98].

Demonstration that the approved drugs Entrectinib and Capivasertib prevent DTT-induced mitochondrial TrkAIII activation and enhance the sensitivity of TrkAIII-expressing SH-SY5Y cells to DTT-induced cell death is of clinical relevance. This is particularly so for Entrectinib, which has been reported to exhibit profound durable efficacy in an infant with refractory advanced-stage metastatic NB, exhibiting TrkAIII expression and activation [99]. Entrectinib use is currently restricted to cancers driven by Trk fusion oncogenes, for which it exhibits profound, durable efficacy [13], supporting calls to extend clinical Entrectinib approval to refractory, advanced-stage metastatic NBs that exhibit TrkAIII expression and activation, bolstered also by evidence that TrkAIII behaves as an oncogenic equivalent to the TrkT3 fusion oncogene in NB models [6] and alternative splicing is a frequent oncogenic pathways activation mechanism in tumors, such as NBs, that exhibit low gene mutation rates [14,15,16,17].

It is worth noting that HA-15, GA, W7, LY29004, Capivasertib, Lestaurtinib and Entrectinib all enhanced DTT-induced killing of TrkAIII SH-SY5Y cells, inhibited in proliferation by DTT, unveiling the potential to promote stress-induced elimination of “dormant” stem cell-like TrkAIII-expressing NB cells [100], removing a potential source of disease progression and post-therapeutic relapse [6,8]. This may help to explain the remarkable durable therapeutic response to Entrectinib reported in an infant with refractory metastatic TrkAIII-expressing NB [99] and also the durable responses to other Trk inhibitors reported in cancers driven by TrkA fusion oncogenes [13]. It is also of note that DTT combined with W7 induced the highest levels of TrkAIII SH-SY5Y cell death (≈90% at 48 h), suggesting that stress, when combined with TrkAIII inhibition and disruption of calmodulin-dependent mitochondrial Ca^2+^ homeostasis, may have the greatest potential to eliminate TrkAIII-expressing NB cells.

## 4. Materials and Methods

### 4.1. Cell Lines and Culture Conditions

Previously described stable transfected empty vector control pcDNA-SH-SY5Y and TrkAIII SH-SY5Y cells [6] were generated from the parental SH-SY5Y NB cell line, which was kindly provided by Dr. U.P. Thorgiersson (NCI, NIH Bethesda, MD, USA). PcDNA-SH-SY5Y and TrkAIII SH-SY5Y cells were routinely cultured at 37 °C and 5% CO_2_, in RPMI 1640, supplemented with 10% fetal bovine serum (Euroclone, Milan, Italy), 1% glutamine (Euroclone, Milan, Italy), 1% penicillin/streptomycin and Zeocin, as a selection antibiotic for stable transfectants (Thermo Fisher Scientific, Waltham, MA, USA).

### 4.2. Reagents and Antibodies

Dithiothreitol (DTT), geldanamycin, Brefeldin A, Lestaurtinib, LY294002, PD98059, Calmodulin-Sepharose^TM^ 4B and protein A Sepharose^TM^ (fast flow) were from Sigma-Aldrich (Saint Louis, MO, USA). HA15, Honokiol Dichloroacetate (Honokiol DCA) and Capivasertib were from Med Chem Express (Monmouth Junction, NJ, USA). Entrectinib was from Selleck Chemicals (Houston, TX, USA), DS16570511 was from Cayman Chemical (Ann Arbour MI, USA), resveratrol was from ENZO Life Sciences Inc (Farmingdale, NY, USA), and W7 was from Millipore Sigma (Burlington, MA, USA). IncuCyte^®^ Cytotox Green Dye (4633) was from Sartorius (Goettingen, Germany). Mouse monoclonal anti-human TrkA (B3, sc-7268), rabbit polyclonal anti-human XIAP (H-202), rabbit polyclonal anti-human α-tubulin (H-3000) and monoclonal mouse anti-human calmodulin (sc-137079) antibodies were from SantaCruz (Santa Cruz, CA, USA). Polyclonal rabbit anti-human Y490 phosphorylated TrkA (9141), polyclonal rabbit anti-human Akt (9272), polyclonal rabbit anti-human phospho-Ser 473-Akt (4060), polyclonal rabbit anti-human Y674/675 (4621), antibody was from Cell Signaling Technology (Danvers, MA, USA). Polyclonal rabbit anti-human GRP78/BiP (ET-24) and anti-human Hsp-60 (HPA001523) antibodies were from Sigma-Aldrich (St Louis, MO, USA). Horseradish peroxidase (HRP)-conjugated goat anti-rabbit and rabbit anti-mouse secondary antibodies were from Bethyl Laboratories Inc. (Fortis, Waltham, MA, USA). Mouse monoclonal SOD2 (611581) and TOM20 (612278) antibodies were from BD Transduction Laboratories (San Jose, CA, USA). Mouse monoclonal anti-human calnexin antibody (ab2798) was from AbCam (Amersham, UK). Mouse monoclonal anti-oxidized PTPases (MAB2844) were from R&D Systems Inc. (Minneapolis, MN, USA). Alexa Fluor 488-labeled donkey anti-rabbit and Alexa Fluor donkey anti-mouse secondary antibodies were from Life Technologies (Waltham, MA, USA). ProLong^TM^ Gold anti-fade reagent with DAPI was from Invitrogen (Thermo Fisher Scientific, Waltham, MA, USA).

### 4.3. In Vitro IncuCyte Cytotoxicity Assays

Cytotoxicity was assessed over a 48 h time course in an IncuCyte^®^ S3 Live-Cell Analysis System incubator, as directed (Sartorius, Goettingen, Germany), using Incucyte^®^ Cytotox Green Dye (4633) for detecting cytotoxic disruption of cell membrane integrity. Cells were seeded at equal densities of 1 × 10^4^ per well in tissue culture-treated 96-well cell culture plates (353072, Corning Inc., New York, NY, USA), allowed to attach for 4 h at 37 °C. Duplicate wells were then treated with either 5 mM DTT alone or with combinations of 5 mM DTT and the various inhibitors at concentrations indicated in figure legends, in the presence of 150 nM Incucyte^®^ Cytotox, formulated to detect cell membrane-integrity disruption in real time to quantify cell death, and experiments were incubated in the IncuCyte^®^ S3 Live-Cell Analyzer, programmed for time-lapse video photography of 2 independent areas per well, at 1 h intervals and at 10× magnification, and cell behaviour was recorded by time-lapse video. For cell-death evaluation (Figure 3c, Figure 4c and Figure 5g), cells exhibiting nuclear uptake and no nuclear uptake of Incucyte^®^ Cytotox Green Dye were directly counted in phase contrast micrographs captured from each individual time-lapse video at 0, 24 h and 48 h. The number of dead cells exhibiting nuclear Green Dye uptake were calculated as a percentage of total cell numbers (cells exhibiting nuclear Green Dye plus cells exhibiting no Green Dye uptake) in each duplicate well. For statistical analysis and graphics, mean (±s.d.) percentage cell death was calculated in 3 independent experiments for each treatment, performed in duplicate. Data were statistically compared by Student’s *t*-test (online at: https://www.graphpad.com). In all DTT co-treatments, cells were pre-incubated with inhibitors for 30 min prior to the addition of DTT in the continuous presence of inhibitors for the times indicated.

### 4.4. Total Protein Extraction

For total cell proteins, cells were cultured to 80–90% confluence on 100 mm tissue culture-treated culture plates (Corning 430167, Corning Inc., New York, NY, USA). Cells were scraped into lysis buffer, on ice (PBS containing 0.5% sodium deoxycholate, 1% NP40, 0.1% SDS, 1 mM sodium orthovanadate, 1 mM PMSF, 1 µg/mL of pepstatin A, and Aprotinin). Cells were washed in PBS at 4 °C and scraped on ice into 2–3 mL of ice-cold cell lysis buffer. Cell lysates were centrifuged at 15,000× *g* at 4 °C to remove insoluble material, and protein concentrations were determined by the Bradford protein assay, as directed (Thermo Fisher Scientific, Waltham, MA, USA). Protein extracts were resuspended in either denaturing 62.5 mM Tris HCl pH 6.8, 2.5% SDS, 0.002% bromophenol blue and 10% glycerol) or reducing (62.5 mM Tris HCl pH 6.8, 2.5% SDS, 0.002% bromophenol blue, 5% β-mercaptoethanol and 10% glycerol) SDS-PAGE sample buffer. In all DTT plus inhibitor co-treatments, cells were pre-incubated with inhibitors for 30 min prior to addition of DTT in the continuous presence of inhibitors for the times indicated.

### 4.5. Mitochondrial Purification and Extraction

For mitochondrial purification, cells were cultured to 80–90% confluence on 150 mm tissue culture-treated cell culture dishes (Corning 353025, Corning Inc., New York, NY, USA). Mitochondria were purified using a Focus Sub Cell Mitochondrial isolation kit (G-Biosciences, St. Louis, MO, USA), as previously described [20,101]. Briefly, cells were harvested in ice-cold PBS by scraping and centrifuged at 1200× *g* for 5 min at 4 °C. A total of 500 μL of ice-cold kit Buffer I, containing 1× protease inhibitor cocktail (Sigma, St. Louis, MO, USA), was added to cell pellets, and cells were disrupted by 10 passages through a 20-gauge needle. A total of 250 μL of kit Buffer II was then added to the homogenate, and samples were centrifuged at 1200× *g* for 5 min at 4 °C. Supernatants were transferred to fresh tubes, centrifuged at 15,000× *g* for 10 min at 4 °C, and resulting mitochondrial-rich pellets were then washed with 500 μL of kit Buffer II, centrifuged at 15,000× *g* for 10 min at 4 °C and re-suspended in mitochondrial storage buffer (250 mM mannitol, 5 mM HEPES pH 7.4 and 0.5 mM EGTA), layered onto a 30% Percoll gradient in storage buffer and subjected to ultra-centrifugation at 90,000× *g* for 40 min at 4 °C. Ultrapure mitochondria, located 2/3 rds from the top of Percoll gradients, were collected into fresh tubes, diluted 1:10 in 250 mM mannitol, 5 mM HEPES (pH 7.4) containing 0.5 mM EGTA and re-centrifuged at 15,000× *g* for 10 min, at 4 °C in an Eppendorf microfuge. Resulting mitochondrial pellets were lysed in PBS containing 0.5% sodium deoxycholate, 1% NP40, 0.1% SDS, 1 mM sodium orthovanadate, 1 mM PMSF, 1 µg/mL of pepstatin A and Aprotinin and re-centrifuged at 15,000 rpm for 2 min at 4° in an Eppendorf microfuge C to remove insoluble material. Mitochondrial protein concentrations in supernatants were determined by the Bradford protein assay (Thermo Fisher Scientific, Waltham MA, USA) and appropriate concentrations were analysed by reducing SDS-PAGE Western blotting. Mitochondrial purity was checked for translocate of the outer membrane 20 (TOM20), superoxide dismutase 2 (SOD2), heat shock protein 60 (HSP60) and x-linked inhibitor of apoptosis protein (XIAP) positivity relative to calnexin and α-tubulin in comparison to mitochondria-depleted and total TrkAIII SH-SY5Y extracts by Western blotting (Supplementary Figure S1) and [20]. In all DTT co-treatments, cells were pre-incubated with inhibitors for 30 min prior to the addition of DTT in the continuous presence of inhibitors for the times indicated.

### 4.6. Western Blotting

Proteins in total and mitochondrial extracts were denatured and/or reduced at 95 °C for 5 min, separated by SDS-PAGE and then transferred to Hybond C-Extra nitrocellulose membranes (GE Healthcare Life Science, Amersham, UK) and air-dried. Non-specific protein binding sites on air-dried membranes were then blocked by 1 h incubation at room temperature with blocking solution (5% non-fat milk in 15 mM NaCl, 10 mM Tris-HCl, 10% Tween 20, pH 8.0), and blocked membranes were then incubated overnight with primary antibodies diluted in blocking solution at 4 °C with oscillation. Primary antibody dilutions were 1:250 for anti-oxidized PTPases; 1:500 for the anti-TrkA antibody; 1:1000 for anti-Y490 phosphorylated TrkA, anti-Y674/Y675 phosphorylated TrkA, anti-phosphorylated Ser-473 Akt, anti-calmodulin, anti-GRP78, anti-α-tubulin and anti-XIAP antibodies; 1:2000 for anti-SOD2 antibody; 1:3000 for anti-Akt antibody (9272); 1:4000 for anti-calnexin antibody and 1:5000 for anti-TOM20 antibody. Membranes were then washed in TBST solution and incubated with either horseradish peroxidase (HRP)-conjugated goat anti-rabbit or rabbit anti-mouse antibodies diluted 1:2000 in blocking solution for 1 h at room temperature and washed extensively in TBST at room temperature. Immunoreactivity in Western blots was revealed using an Amersham ECL Western blotting detection kit, as directed (Cytiva, Amersham, UK) in an ImageQuant™ LAS 4000 (GE Healthcare, Chicago, IL, USA) image analyser, and Jpeg images were analysed by ImageJ free software (https://imagej.net/ij/download.html, accessed on 14 May 2024) downloaded for routine computer use. Western blots were performed in duplicate, and experiments were repeated a minimum of twice. For all DTT co-treatments, cells were pre-incubated with inhibitors for 30 min prior to the addition of DTT in the continuous presence of inhibitors for the times indicated.

### 4.7. Calmodulin-Sepharose Pulldown

TrkAIII SH-SY5Y and pcDNA-SH-SY5Y cell extracts in lysis buffer (Section 4.4) were prepared from cells grown to 80-90% on 100 mm tissue culture-treated culture plates (Corning 430167, Corning Inc., NY, USA). CaM-Sepharose^TM^ 4B (Sigma-Aldrich, Saint Louis, MO, USA) was blocked in PBS containing 1% BSA for 1 h at 4 °C with rotation, added to cell extracts in lysis buffer (500 µg) and incubated for 2 h at 4 °C with rotation in the presence of either 100 µM CaCl_2_ or 2 mM EGTA. Following incubation, CaM-Sepharose^TM^ 4B conjugates were centrifuged at 15,000 rpm in an Eppendorf centrifuge, resuspended and washed three times with 500 µL of Triton X-100 buffer (20 mM Tris HCl pH 7.5, 150 mM NaCl, 1% Triton X-100), containing either CaCl_2_ or EGTA. Final CaM-Sepharose^TM^ 4B conjugated pellets were resuspended in SDS-PAGE reducing sample buffer, heated to 95 °C for 5 min and analysed by reducing SDS-PAGE Western blotting.

### 4.8. Co-Immunoprecipitation

TrkAIII SH-SY5Y cells grown to 80–90% on 100 mm tissue culture-treated culture plates (Corning 430167, Corning Inc., NY, USA) were either untreated or treated for 6 h with 5 mM DTT in complete culture medium, then lysed in lysis buffer (Section 4.4). Cell lysates in lysis buffer (1 mg) were pre-cleared by 2 h incubation with 1 µg/mL of pre-immune IgG and 20 µL/mL of pre-washed fast flow protein A Sepharose at 4 °C with rotation. IgG bound protein A Sepharose was removed by centrifugation at 15,000 rpm for 5 min at 4 °C in an Eppendorf microfuge, and pre-cleared extracts were then incubated overnight with anti-CaM or anti-TrkA antibodies, diluted to 1:200 in lysis buffer at 4 °C with rotation. Following overnight incubation, 20 µL of protein A Sepharose in cell lysis buffer was added to reactions and incubated for 1 h at 4 °C with rotation. Protein A Sepharose/antibody conjugates were then collected by centrifugation (15,000 rpm for 5 min) in an Eppendorf microfuge, washed in 1 mL of lysis buffer, re-centrifuged and reduced in SDS-PAGE reducing buffer (62.5 mM Tris HCl pH 6.8, 2.5% SDS, 0.002% bromophenol blue, 5% β-mercaptoethanol and 10% glycerol). Samples were then subjected to reducing SDS-PAGE Western blotting. The immunoreactivity of CaM, GRP78/BiP and TrkAIII was detected using anti-CaM (1:1000 dilution), GRP78-BiP (1:1000 dilution) and anti-TrkA (1:500 dilution) antibodies and was compared to appropriate pre-immune IgG immunoprecipitation controls. These experiments were performed in duplicate and repeated.

### 4.9. Indirect Immunofluorescence

The cells were grown to sub-confluence on Nunc glass chamber slides (Nunc Lab-Tek II Chamber Slide system, Sigma-Aldrich, St Louis, MO, USA) and were either untreated or treated with 5 mM DTT for 6 h. At 6 h, cell cultures were washed three times in PBS, fixed in formalin 10% *v*/*v* and permeabilized in 100% ice-cold methanol (−20 °C) overnight. Fixed, permeabilized cells were incubated for 1 h in blocking solution (1% bovine serum albumin in PBS-0.03% TX100), then incubated for 2 h with primary anti-TrkA or anti-Y490 phosphorylated TrkA antibodies (1:100 dilution) in blocking solution at room temperature. Following incubation, slides were washed three times in PBS-0.03% TX100 and incubated with secondary fluorochrome-conjugated Alexa Fluor 488-labeled donkey anti-rabbit or Alexa Fluor donkey anti-mouse antibodies, diluted to 1:500 in blocking solution, for 1 h at room temperature. Following incubation, slides were washed extensively in PBS, mounted with ProLongTM Gold anti-fade reagent, containing DAPI nuclear stain, observed under a Zeiss Axioplan 2 fluorescence microscope and digitally photographed. For this study, IF experiments were performed in duplicate and repeated 3 times.

### 4.10. Densitometry, Statistical Analysis and Software

Data were analysed by Student’s *t*-test using the online *t*-test calculator at https://www.graphpad.com/quickcalcs/ttest1.cfm, accessed on 14 May 2024, accessed routinely over the course of this work, with significance differences associated with probabilities of ≤0.05. Mitochondrial TrkAIII translocation probability was calculated using the online protein N-terminal mitochondrial translocation probability calculator at http://mitf.cbrc.jp/MitoFates/cgi-bin/top.cgi, accessed 1 May 2017 [20].

## 5. Conclusions

This pro-survival adaptation of the integrated stress response that mitigates the cytotoxicity of stress-induced disruption of mitochondrial Ca^2+^ homeostasis in TrkAIII-expressing SH-SY5Y NB cells is relevant to TrkAIII-expressing NB cell selection within the stressful tumor microenvironment and helps to explain the correlation reported between alternative TrkAIII splicing, advanced-stage metastatic disease and post-therapeutic relapse reported in human NBs [6,8]. The altered behaviour of TrkAIII that underpins this mechanism depends upon TrkAIII misfolding and complexing with Grp78 and Ca^2+^-calmodulin, which combined with Hsp90 and Arf-1 regulate mitochondrial importation of misfolded TrkAIII into IMMs. IMM-associated misfolded TrkAIII N-termini are then degraded by the IMS protease Omi/HtrA2, resulting in the accumulation of correctly folded functional TrkAIII C-termini in mitochondrial matrix orientation, which are then activated by a CaMKII/Akt-dependent mechanism, involving the MCU and ROS, in association with mitochondrial PTPase oxidation. Activated IMM-associated TrkAIII augments mitochondrial Akt activation via PI3K, confirming a stress-induced pro-survival mitochondrial TrkAIII–PI3K–Akt axis. We propose that misfolded TrkAIII represents a novel stress-induced Ca^2+^-calmodulin- and CaMK-regulated mitochondrial kinase in addition to a novel pro-survival component of the integrated ER–mitochondrial stress response involved in mitigating the cytotoxicity of the stress-induced disruption of mitochondrial Ca^2+^ homeostasis. We also identify Grp78, Ca^2+^-calmodulin, Hsp90, PI3K and Akt as novel targetable participants in this mechanism, the inhibition of which enhances stress-induced killing of TrkAIII-expressing NB cells, and characterize Arf1, the MCU and ROS as non-targetable participants due to their direct involvement in DTT-induced death. We propose that these novel targets are of potential therapeutic relevance to enhancing stress-induced elimination of TrkAIII-expressing NB cells involved in disease progression and post-therapeutic relapse.

## Figures and Tables

**Table 1:** Inhibitor effects on DTT-induced mitochondrial TrkAIII cleavage, mitochondrial TrkAIII and Akt phosphorylation, proliferation and DTT-induced death in pcDNA-SH-SY5Y and TrkAIII SH-SY5Y cells.

Inhibitor	Target	TrkAIII Cleavage Inhibition	TrkAIII Phos Inhibition	pcDNA- SH-SY5Y Akt Phos Inhibition	TrkAIII SH-SY5Y Akt Phos Inhibition	pcDNA- SH-SY5Y Division	TrkAIII SH-SY5Y Division	DTT-Induced pcDNA- SH-SY5Y Death	DTT-Induced TrkAIII SH-SY5Y Death
**HA-15**	**Grp78**	Y	Y	nd	nd	N	N	N	>
**W7**	**CaM**	Y	Y	nd	nd	Y	Y	N	>
**BrefeldinA**	**Arf1**	Y	Y	nd	nd	Y	Y	N	<
**Geldanamycin**	**Hsp90**	y	Y	nd	nd	Y	Y	N	>
**Honokiol DCA**	**TRAP1**	N	N	nd	nd	N	N	N	N
**DS16570511**	**MCU**	N	Y	nd	nd	Y	Y	N	<
**Resveratrol**	**ROS**	y	Y	nd	nd	Y	Y	N	<
**Entrectinib**	**Trk**	N	Y	N	Y	N	N	N	>
**Lestaurtinib**	**Trk**	N	Y	nd	nd	Y	Y	N	>
**Capivasertib**	**Akt**	N	Y	nd	N	Y	Y	N	>
**LY294002**	**PI3K**	N	N	N	Y	Y	Y	N	>
**PD098059**	**MAPK**	N	N	N	N	N	N	N	N
**KN-93**	**CAMK**	N	Y	Y	Y	nd	nd	nd	nd
**STO-609**	**CAMKK**	N	N	N	N	nd	nd	nd	nd

Y = complete inhibition; y = partial inhibition; N = no effect; < = decrease; > = increase; nd = not done.

## Data Availability

The data presented in this study are available from the corresponding author upon reasonable request.

## Supplementary Materials

The following supporting information can be downloaded at https://www.mdpi.com/article/10.3390/ijms25105475/s1.
