# Supplementary figures and images for: Molecular Characterization and Inhibition of a Novel Stress-Induced Mitochondrial Protecting Role for Misfolded TrkAIII in Human SH-SY5Y Neuroblastoma Cells
Source: Int J Mol Sci. 2024 May 17;25(10):5475. doi: 10.3390/ijms25105475 (PMC11122047; doi:10.3390/ijms25105475)

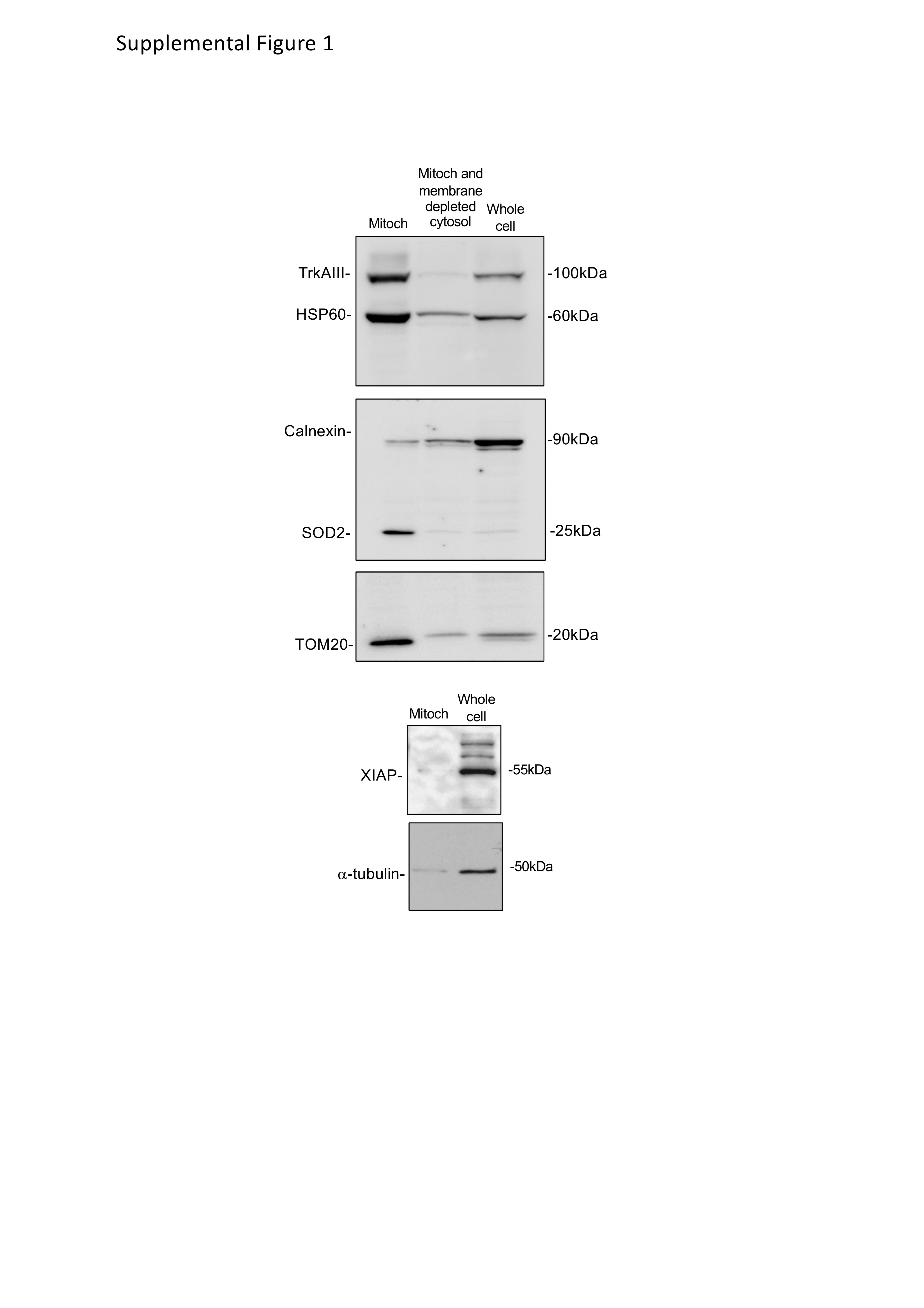

Supplement: Supplementary file 1 [file ijms-25-05475-s001.zip › Figure S1.jpg]
